# Human serum-derived α-synuclein auto-antibodies mediate NMDA receptor-dependent degeneration of CNS neurons

**DOI:** 10.1186/s12974-024-03050-6

**Published:** 2024-02-28

**Authors:** Pretty Garg, Franziska Würtz, Fabian Hobbie, Klemens Buttgereit, Abhishek Aich, Kristian Leite, Peter Rehling, Sebastian Kügler, Mathias Bähr

**Affiliations:** 1 Department of Neurology, University Medical Center Göttingen, Waldweg 33, 37073 Göttingen, Germany; 2 Cluster of Excellence “Multiscale Bioimaging: from Molecular Machines to Networks of Excitable Cells” (MBExC), University of Göttingen, 37073 Göttingen, Germany; 3 Department of Cellular Biochemistry, University Medical Center Göttingen, Göttingen, Germany

**Keywords:** α-synuclein, Auto-antibodies, Neurons, Astrocytes, Neurodegeneration, Network activity, Calcium, NMDA receptor, Parkinson´s disease

## Abstract

**Background:**

Presence of autoantibodies against α-synuclein (α-syn AAb) in serum of the general population has been widely reported. That such peripheral factors may be involved in central nervous system pathophysiology was demonstrated by detection of immunoglobulins (IgGs) in cerebrospinal fluid and brain of Parkinson’s disease (PD) patients. Thus, blood-borne IgGs may reach the brain parenchyma through an impaired blood-brain barrier (BBB).

**Findings:**

The present study aims to evaluate the patho-physiological impact of α-syn AAbs on primary brain cells, i.e., on spontaneously active neurons and on astrocytes. Exposure of neuron-astrocyte co-cultures to human serum containing α-syn AAbs mediated a dose-dependent reduction of spontaneous neuronal activity, and subsequent neurodegeneration. Removal specifically of α-syn AAbs from the serum prevented neurotoxicity, while purified, commercial antibodies against α-syn mimicked the neurodegenerative effect. Mechanistically, we found a strong calcium flux into neurons preceding α-syn AAbs-induced cell death, specifically through NMDA receptors. NMDA receptor antagonists prevented neurodegeneration upon treatment with α-syn (auto)antibodies. α-syn (auto)antibodies did not affect astrocyte survival. However, in presence of α-syn, astrocytes reacted to α-syn antibodies by secretion of the chemokine RANTES.

**Conclusion:**

These findings provide a novel basis to explain how a combination of BBB impairment and infiltration of IgGs targeting synuclein may contribute to neurodegeneration in PD and argue for caution with α-syn immunization therapies for treatment of PD.

**Supplementary Information:**

The online version contains supplementary material available at 10.1186/s12974-024-03050-6.

## Background

Parkinson’s disease (PD) is one of the most common neurodegenerative disorders, affecting ten million people worldwide, constituting 1% of the population above the age of 65 years and 5% above the age of 85 [1]. PD is classically diagnosed with the onset of motor symptoms like rigidity, resting tremor, bradykinesia, and postural instability [2]. These symptoms are primarily caused by the loss of dopaminergic neurons in substantia nigra pars compacta [3–5]. However, non-motor symptoms (NMS) like fatigue, pain, depression, dementia, loss of smell, and sleep fragmentation are also frequently reported among PD patients. A huge phenotypic variability exists in NMS as well as in motor symptoms, and in non-motor fluctuation (NMF) after chronic levodopa therapy [6–8]. Factors such as demographics, pathological changes, and genetics only partially address the symptomatic heterogeneity in PD [9], whereas the basis for intraindividual NMS and NMF variability remains largely enigmatic.

If and how phenotypic variability relates to extent of aggregation of the pre-synaptic protein α-syn, which constitutes a major component of the Lewy bodies/neurites found as pathological hallmarks in Parkinsonian brains [10], is also unknown. Extent of aggregation and potential spread of pathological strains of α-syn are not well correlated to clinical phenotypes of PD [11, 12]. None the less, α-syn and its aggregated forms are prominent targets for therapy approaches trying to reduce their impact on the brain, e.g. by active and passive immunization against this highly abundant protein [13]. However, α-syn appears to be naturally addressed by the human immune system. A recent study has shown T-cell responses to N- and C-terminal peptides of α-syn in about 40% of the studied PD patients [14]. A plethora of studies reported the presence of autoantibodies (AAb) against α-syn in the serum of healthy subjects and PD patients [15, 16]. Lately, it has been shown that independent of their age and sex, almost all individuals present circulating α-syn AAbs [17]. These naturally occurring anti-α-syn AAbs have been shown to bind to both monomers and oligomers of α-syn [18, 19]. Additionally, several reports support the notion of a leaky blood-brain barrier (BBB) in PD patients. Increased level of albumin and immunoglobulin G (IgG) in the cerebro-spinal fluid (CSF) and a reduced P-glycoprotein function in the midbrain of PD patients has been previously reported [20, 21]. Importantly, the presence of IgGs was demonstrated in post-mortem brain tissue on neurons specifically in the midbrain region of idiopathic and familial PD patients, but not in controls [22]. It is currently unknown if in PD these IgGs are solely derived from periphery, or might also be produced intrathecally from infiltrated B-cells as has been suggested for certain neuroinflammatory conditions [23–25].

Considering a compromised BBB in PD patients, invasion of the brain by circulating α-syn AAbs is very likely. So far, α-syn AAbs were considered to act potentially neuroprotective, as they are able to modify the aggregation propensities of α-syn, and did not show any toxicity when applied to cell lines [18, 19, 26, 27]. In the study presented here, we address the impact of α-syn AAbs present in the serum of healthy subjects and PD patients on the survival of primary rat neurons and astrocytes. We found an NMDA receptor-dependent cell death in neurons, while astrocytes in the presence of α-syn reacted to α-syn AAbs with potentially neuroprotective cytokine secretion. These findings are of relevance not only to advance our understanding of the potential role of the immune system in PD but also to recognize prospective challenges associated with α-syn immunotherapies, be they passive [13] or active [28].

## Results

### α-synuclein autoantibodies cause neurodegeneration of α-synuclein expressing neurons in a dose-dependent manner

**Fig. 1:**
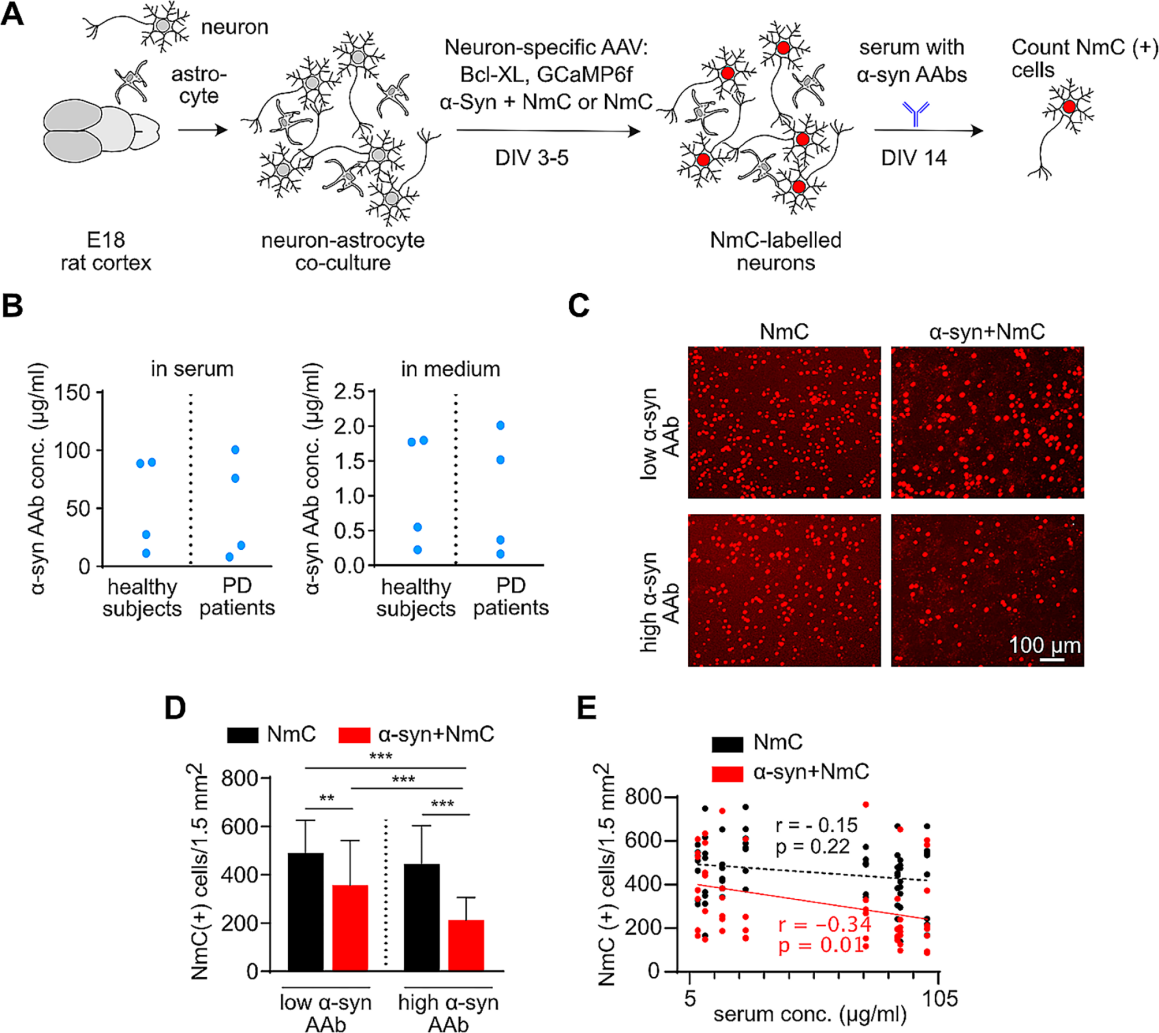
α-synuclein autoantibodies cause a dose-dependent loss of α-synuclein overexpressing neurons. **(A)** Experimental outline showing generation of neuron-astrocyte co-cultures. Cells were transduced with adeno-associated viral (AAV) vectors encoding Bcl-XL and the calcium sensor GCaMP6f under control of the strictly neuron-specific synapsin1 promoter. Additionally, cells were transduced with bi-cistronic AAV vectors encoding α-synuclein (α-syn) and the fluorescent label nuclear-targeted mCherry (NmC) from independent, neuron-specific transcription units, or expressing only NmC as control. At DIV 14, cells were treated with human serum containing α-syn autoantibodies (α-syn AAb). **(B)** Concentration and range of α-syn AAbs in human serum (left panel) and in culture medium (right panel). A total of 8 serum samples were used. Depending on the concentration of the α-syn AAbs, serum samples were categorized as “low” or “high”, each with 4 samples obtained from 2 healthy subjects and 2 PD patients. **(C)** Live imaging of NmC - positive cells in α-syn + NmC or NmC only transduced cultures after 1 d of exposure with serum-containing low (upper panel) or high levels (lower panel) of α-syn AAbs. **(D)** Neuronal survival represented by the quantification of NmC-positive cells after 1 d of exposure to serum-containing low or high levels of α-syn-AAbs. **(E)** Correlation analysis between serum concentration and surviving NmC-expressing cells upon serum treatment. A significant, negative correlation was observed in α-syn + NmC overexpressing cells treated with serum. *N* = 8–10 biological and 32–40 technical replicates for **(D, E)**. Statistics by one-way ANOVA with Tukey’s multiple comparisons test; statistical power (1-ß error probability) > 0.9 for all conditions. ** = *p* < 0.01; *** = *p* < 0.001

To evaluate physiological effects of α-syn AAbs on primary neurons in neuron-astrocyte co-culture (Fig. 1A), we used a total of 8 serum samples from healthy subjects and PD patients, derived from a large and well characterized collection of serum samples [29]. We selected serum samples within either low or high range of the reported α-syn AAb levels (Fig. 1B, left panel). The final concentration of the α-syn AAbs in the cell culture medium ranged between 0.16 and 2.0 µg/ml (Fig. 1B, right panel). Studies were performed in well-established rat primary neuron-astrocyte coculture, where human α-syn plus the fluorescent label nuclear mCherry (NmC), or NmC alone, was expressed specifically in neurons. The fluorescent label served as a reliable means to quantify living neurons, occasionally verified by immunocytochemistry. Cell cultures were maintained without medium exchange to allow the extracellular accumulation of naturally secreted α-syn [30]. Any cell death due to α-syn expression was prevented by low-level expression of the anti-apoptotic protein, Bcl-XL [31].

At DIV 14, cells were treated with serum samples containing different concentrations of α-syn AAbs. Stability of α-syn AAbs in the medium at 37 °C was tested using ELISA for up to 7 days (Suppl. Figure S1). The number of surviving neurons was determined by counting NmC-expressing cells. A significant loss of neurons was observed within 1 day of serum treatment specifically in α-syn expressing cultures. Upon segregating the serum samples containing a low or high level of α-syn AAbs, the extent of neurotoxicity was found to be dependent on the α-syn AAb concentration (Fig. 1C and D; Suppl. Fig. S2). The dose-dependent neurotoxicity of α-syn AAbs was further confirmed by a correlation analysis. The concentration of α-syn AAbs present in the serum showed a significant inverse correlation with the surviving NmC-positive cells 1 day after treatment (Fig. 1E). In the control condition with only NmC expression, no neurodegeneration was observed even after treatment with the serum containing the highest levels of α-syn AAbs. The degree of toxicity caused by serum obtained from healthy subjects and PD patients was not significantly different (neuron numbers / 1.5 mm^2^ at 3 days after adding sera: healthy subjects = 391.8 +/- 52.3, *n* = 40; PD patients = 332 +/- 41, *n* = 28; 2-tailed T-test *p* = 0.41; mean +/- SEM). Therefore, due to the ease of availability of serum, we performed further experiments with a sample obtained from a healthy subject, using a concentration of 1 µg/ml of α-syn AAbs, which resulted in robust neurodegeneration (Suppl. Fig S2.)

**Fig. 2:**
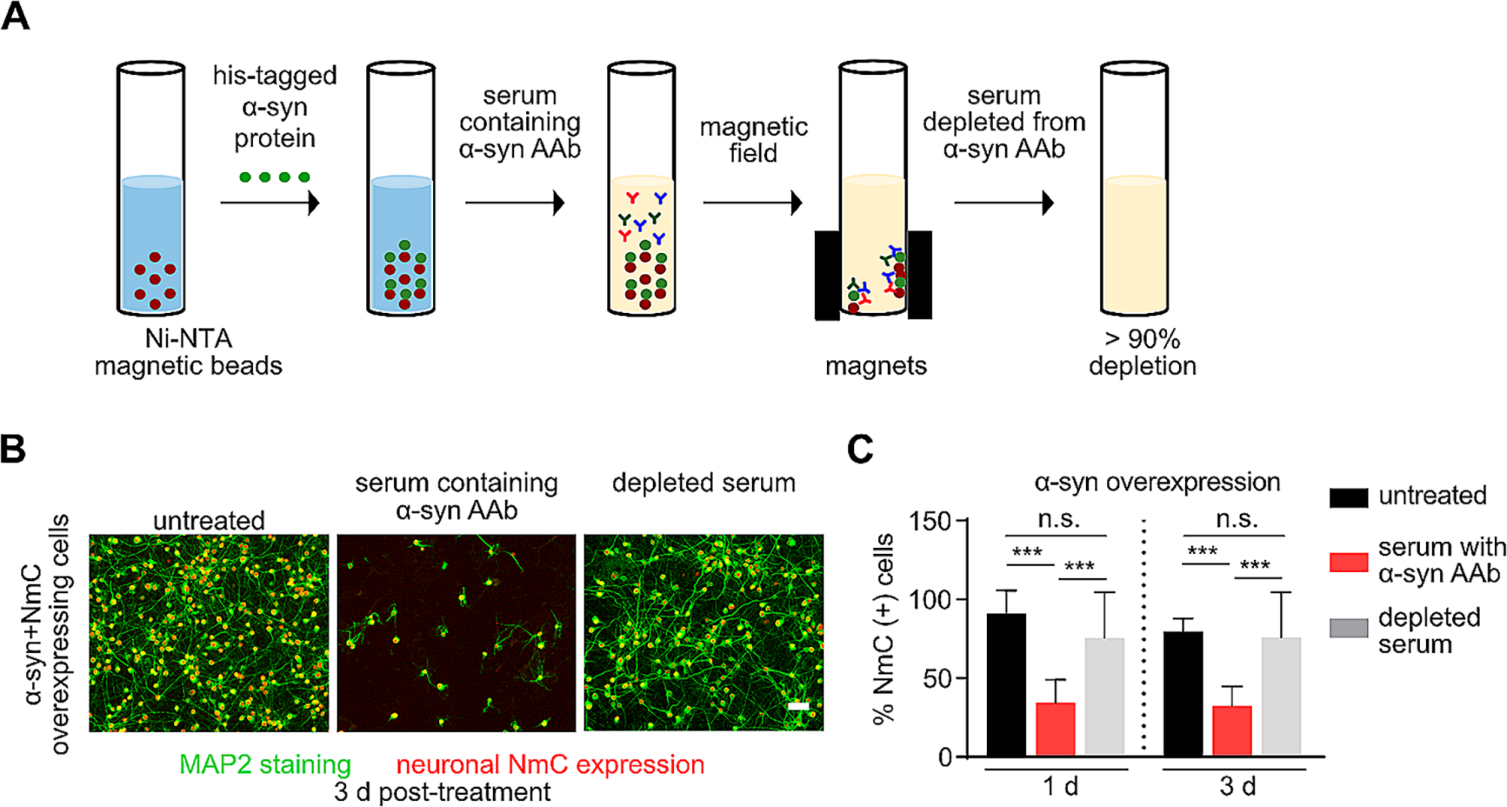
Removal of α-synuclein autoantibodies from serum rescues neurotoxicity. **(A)** Schematic representation of removal of α-syn AAbs from serum. **(B)** Representative photomicrographs of MAP2 staining (green) and NmC-positive cells (red) in α-syn + NmC transduced cultures, either without treatment (untreated), or after 1 d of exposure to serum containing α-syn AAbs, or to depleted serum. **(C)** Percent NmC positive cells (relative to untreated cells) after 1 and 3 d of exposure to serum containing α-syn AAbs or depleted serum, in α-syn + NmC expressing cells. *N* = 4 biological and 8 technical replicates for **(C)**. Statistics by one-way ANOVA with Tukey’s multiple comparisons test; statistical power (1-ß error probability) > 0.9 for all conditions. n.s. = not significant; *** = *p* < 0.001

We next confirmed that neurotoxicity was indeed mediated by α-syn AAbs and not by other factors present in the serum. To this end, we depleted α-syn AAbs from the serum by incubating the serum with α-syn protein bound to magnetic beads (Fig. 2A). Depletion of the α-syn AAbs from the serum was confirmed by ELISA (Suppl. Fig S3). Upon treating the cells with serum depleted from α-syn AAbs, the number of NmC positive cells was not significantly different from untreated control neurons, even at the prolonged timepoint of up to 3 days (Fig. 2B, C). Altogether, these data show that serum containing α-syn AAbs triggers a significant dose-dependent loss of α-syn-expressing neurons. Removal of α-syn AAbs from the serum rescues its neurotoxic effects, proving that specifically α-syn-binding AAbs are responsible for induction of neurodegeneration.

### Antibodies against α-synuclein monomers exert neurotoxicity similar to α-synuclein autoantibodies

**Fig. 3:**
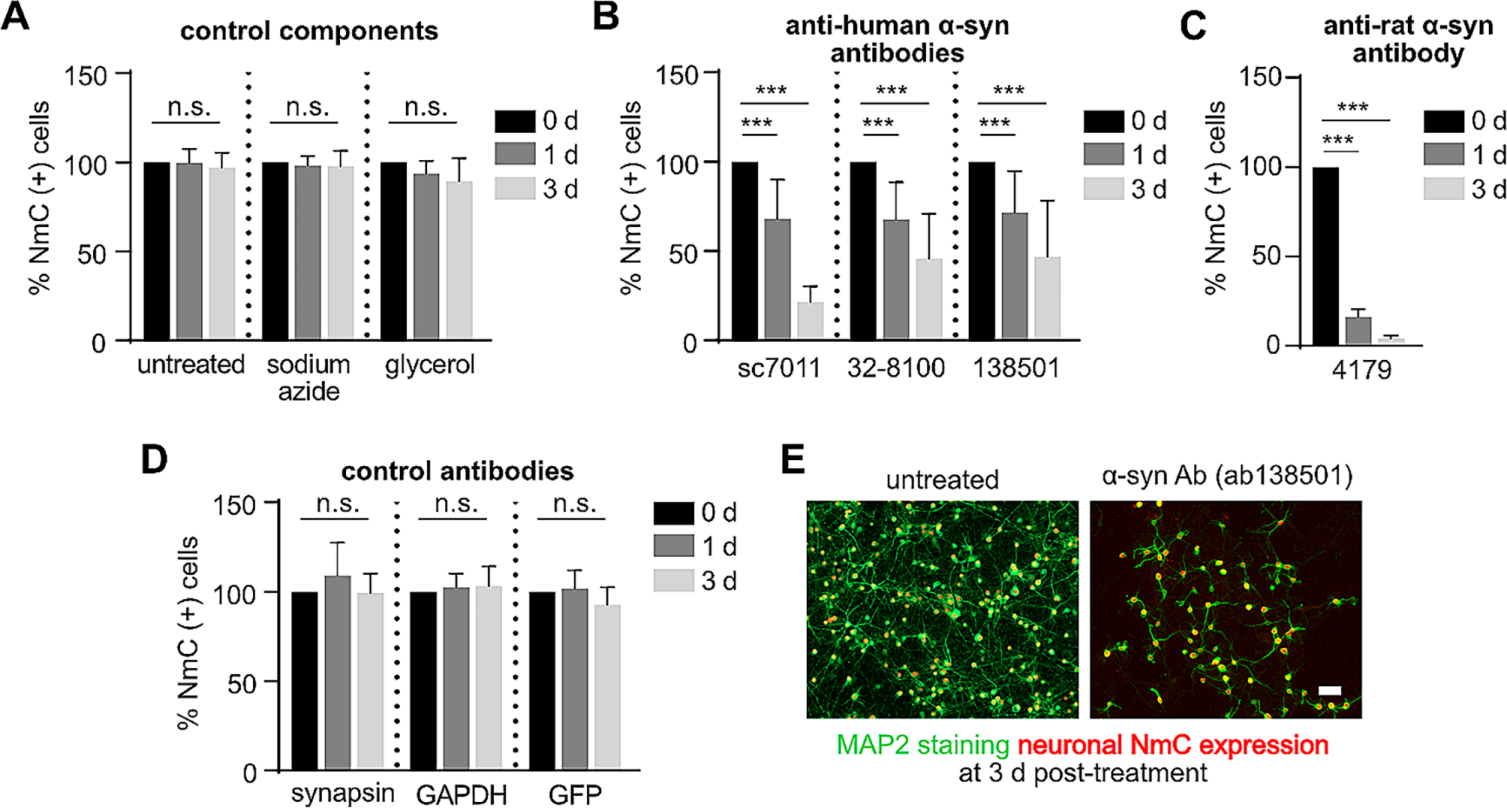
Purified, commercial α-synuclein antibodies exert similar neurotoxic effects as observed with serum containing α-synuclein autoantibodies. **(A)** Percent NmC-positive cells, relative to untreated (pre-treatment) 0 d, upon exposure to antibody vehicle controls, sodium azide and glycerol. **(B)** Percent NmC-positive cells, relative to pre-treatment at 0 d, at 1 d and 3 d after treatment with several commercial anti-human α-syn Abs. **(C)** Percent NmC-positive cells, relative to pre-treatment at 0 d, at 1 d, and 3 d after treatment with rat-specific α-syn antibody. Note: cells used for (C) did not overexpress human α-syn, but only NmC. **(D)** Percent NmC-positive cells, relative to pre-treatment at 0 d, at 1 d and 3 d after treatment with antibodies against Synapsin-1, GAPDH, and GFP. **(E)** Representative images of MAP2 staining and neuron-specific NmC expression in α-syn + NmC transduced cells, after 3 d of exposure with α-syn Ab (ab138501). All commercial antibodies were applied at a concentration of 1 µg/ml. *N* = 4–5 biological and 8–10 technical replicates. Statistics by one-way ANOVA with Dunnett’s multiple comparison test; statistical power (1-ß error probability) > 0.9 for all conditions. n.s. = not significant; *** = *p* < 0.001

We further confirmed the specificity of α-syn antibodies to cause neurodegeneration by exposing α-syn-expressing neurons to commercially available, purified anti-human-α-syn antibodies. The specificity of the antibodies was tested by probing them against rat cell lysate, recombinant mouse, and human synuclein proteins (Suppl. Figure S4A). The known epitopes available from the manufacturer are shown in Supplemental Fig. 4B with a comparison of the protein sequence of human, mouse, and rat α-syn protein. Antibodies against another synaptic protein, synapsin1, and against unrelated proteins like GAPDH and eGFP were used as controls. Since most of these antibody formulations contained either glycerol or sodium azide, cells were treated with their equivalent concentration to serve as antibody vehicle controls (Fig. 3A). Treatment with antibody vehicle controls showed a similar percentage of surviving neurons as with the untreated cultures at all tested time points. As observed with serum containing α-syn AAbs, purified commercial antibodies against the human α-syn protein caused a significant loss of neurons expressing human α-syn (Fig. 3B, E). Treatment with antibodies against synapsin1 or unrelated proteins like GAPDH or eGFP caused no significant neurodegeneration at the tested time points of 1d and 3d post-treatment (Fig. 3D). Importantly, massive neurodegeneration was observed when NmC-expressing cells expressing only endogenous rodent synucleins were exposed to an antibody specific for rodent α-syn (Fig. 3C), indicating that neurodegeneration induced by α-syn antibodies does not depend on species or overexpression. Vice versa, antibodies specific for human α-syn did not cause cell death in NmC-expressing cells with only endogenous rodent synucleins (Suppl. Figure S5). Specificity of the approach was also proven by showing that antibodies directed against human γ-syn did not cause neurodegeneration in neurons overexpressing either human α-syn or γ-syn (Suppl. Fig S6). These results demonstrated that specifically antibodies targeting α-syn can induce robust degeneration of the neuronal fraction in neuron-glia co-cultures.

### α-synuclein (auto)antibodies induce neurodegeneration in human neurons

As a “humanized” alternative to primary rat neurons, we also investigated the impact of α-syn AAbs on human glutamatergic neurons, generated from iPSCs. Neurons were transduced with an AAV expressing both human α-syn and the NmC fluorophore. The cells were either treated with serum containing α-syn AAbs, with serum depleted from α-syn AAbs, or with a purified antibody directed against human α-syn. As shown in Suppl. Fig S7, the extent of neurodegeneration induced under these conditions was very similar to that seen in primary rat neurons. Given the fact that rat primary cells showed more robust generation of non-induced, endogenous neuronal network activity, could be used to prepare neuron-astrocyte co-cultures without any further manipulation, and were previously well characterized for their ability to release α-syn into the extracellular space [30, 31] we continued to use these cells for all further experiments.

### α-synuclein (auto)antibodies do not affect astrocyte survival but upregulate RANTES secretion

**Fig. 4:**
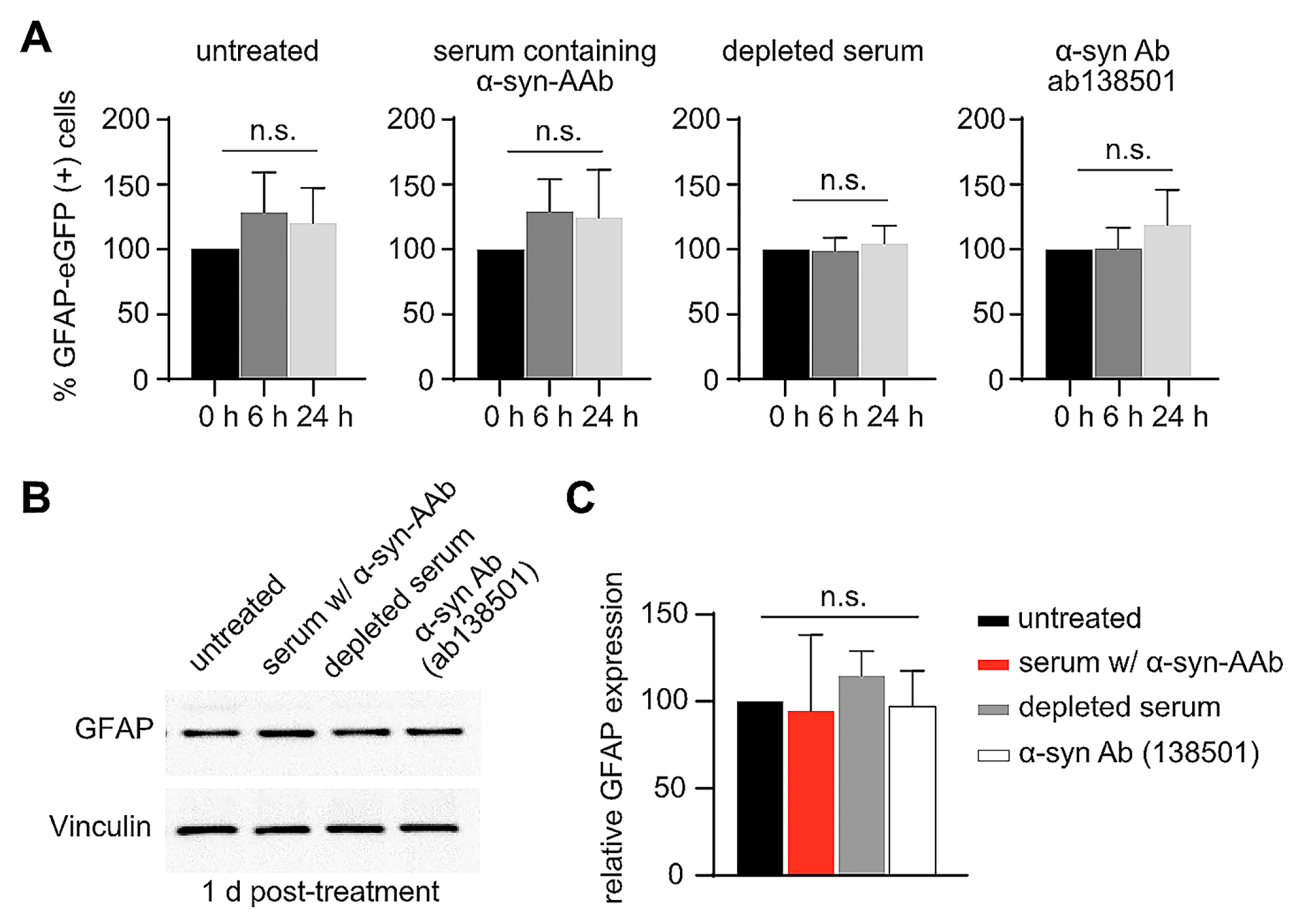
α-synuclein (auto)antibodies do not affect astrocyte survival in rat primary neuron-astrocyte co-cultures. **(A)** Percent GFAP-eGFP positive cells in α-syn + NmC transduced cultures before treatment (0 h) and after 6 and 24 h of treatment with serum containing α-syn AAbs, or with serum depleted of α-syn AAbs, or with an commercial α-syn Ab (ab138501). **(B)** Western blot for astrocyte marker (GFAP) and internal control (Vinculin) with lysates prepared from α-syn + NmC transduced cultures after 24 h of treatment. **(C)** Quantification of western blots as relative GFAP expression after normalization to Vinculin expression. *N* = 4–5 biological replicates for all conditions. Statistics by one-way ANOVA with Dunnett’s multiple comparisons test

The importance of astrocytes for survival, maturation and activity of neurons is widely accepted [32]. Since both astrocytes and neurons are present in our culture system, we questioned whether the application of α-syn (auto)antibodies would affect astrocytes as well. To quantitatively assess astrocyte numbers in living cultures, we used eGFP expression from a strictly astrocyte-specific GFAP promoter [33] to visualize astrocyte survival at 6 and 24 h after treatment with α-syn (auto)antibodies (Suppl. Figure S8A). In these co-cultures, α-syn expression was restricted to neurons as described above. We observed no effect of α-syn (auto)antibodies on astrocyte survival, with no difference in the number of eGFP expressing cells between untreated and treated conditions (Fig. 4A, Suppl. Figure S8B). We also observed no change in total GFAP expression in any of the treated conditions as a measure of activated astrocytes (Fig. 4B, C).

Our neuron-astrocyte co-culture contained about 10–20% astrocytes at DIV14. To further validate astrocyte survival in the presence of α-syn (auto)antibodies, we generated astrocyte-enriched cultures (> 99% astrocytes) and transduced them with astrocyte-specific AAVs, expressing either eGFP alone or α-syn + eGFP. Thus, under these conditions, human α-syn is expressed within astrocytes. Cells were then treated with α-syn (auto)antibodies (Suppl. Figure S9A). As observed with neuron-astrocyte mixed cultures, there was no change in the number of eGFP expressing astrocytes or the total GFAP expression in any of the treated conditions in astrocyte-enriched cultures (Suppl. Figure S9B-D). These data suggest that α-syn (auto)antibodies neither affect astrocyte survival nor enhance GFAP expression, a property of reactive astrocytes [34].

**Fig. 5:**
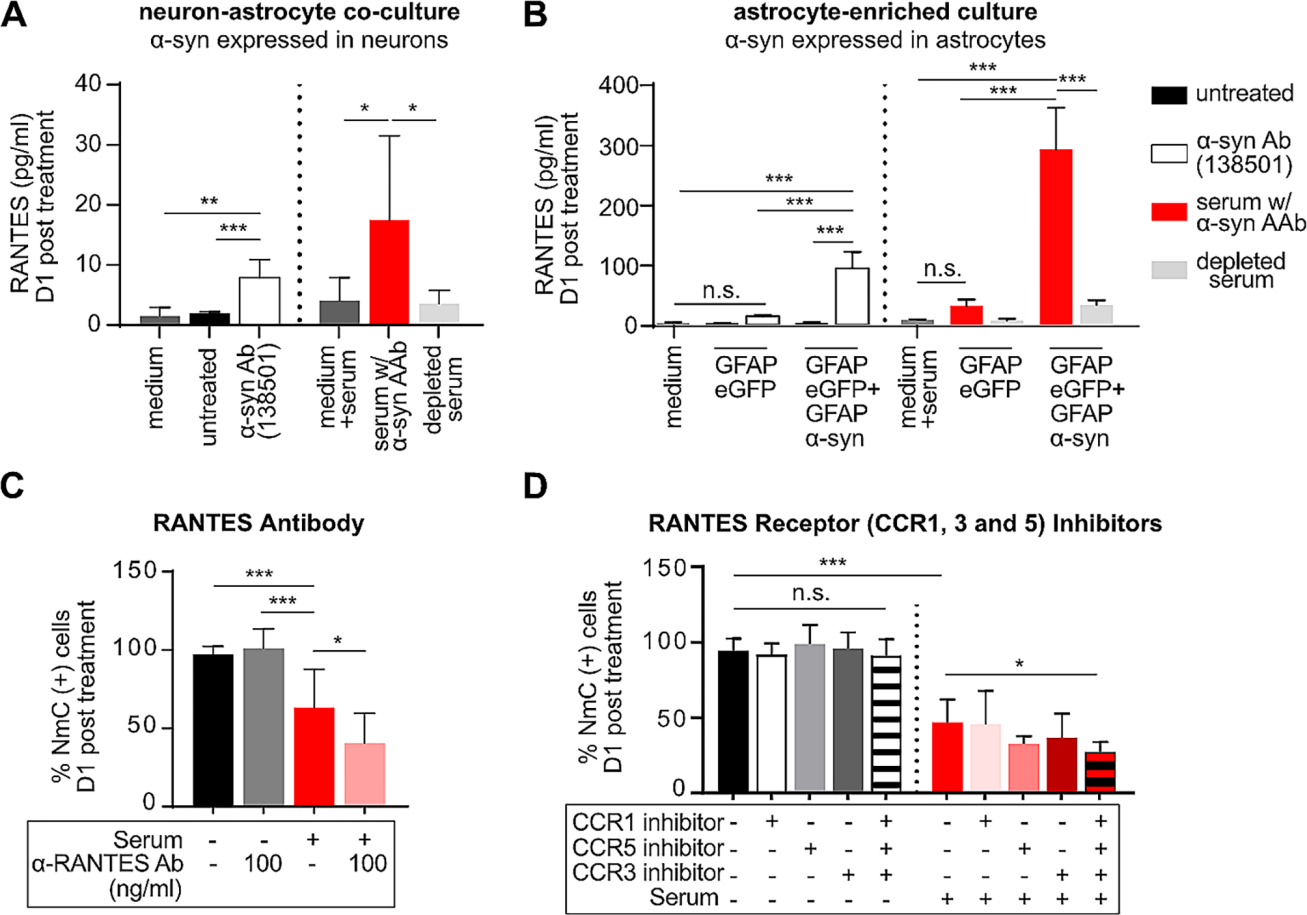
Exposure to α-synuclein (auto)antibodies activates RANTES secretion by astrocytes. **(A)** RANTES levels in cell culture supernatant of neuron-astrocyte co-cultures, with neurons expressing α-syn + NmC. RANTES was quantified at 24 h after treatment of cultures with antibodies or sera at DIV 14. Cultures were either left untreated, or were treated with a commercial anti α-syn Ab (ab138501), or with serum containing α-syn AAbs, or with serum that was depleted for α-syn AAbs. Quantification from pure cell culture medium (“medium”), and from cell culture medium plus serum (“medium + serum”) served as additional background controls. **(B)** RANTES levels in cell culture supernatant of astrocyte-enriched cultures (> 99% astrocytes). α-syn + EGFP or EGFP alone were expressed in astrocytes. RANTES was quantified at 24 h after treatment with the commercial anti α-syn antibody (ab138501), or with serum containing α-syn AAbs or with serum that was depleted for α-syn AAbs. **(C)** Percent NmC-positive cells (relative to untreated controls) upon treatment with serum containing α-syn AAb with or without anti-RANTES antibody. **(D)** Percent NmC-positive cells (relative to untreated controls) upon treatment with α-syn AAb containing serum with or without blockers of RANTES receptors CCR1, 3, and 5. (C) and (D) were obtained in neuron-astrocyte co-cultures. *N* = 4–6 biological replicates for all conditions. Statistics by one-way ANOVA with Tukey’s (**A** – **C**) or Dunnett’s (**D**) multiple comparison test; statistical powers (1-ß error probability): **A**) **, *** > 0.9, * 0.5; **B**) > 0.9 for all conditions; **C**) **, *** > 0.9; * 0.65; **D**) > 0.9 for all conditions. * = *p* < 0.05; ** = *p* < 0.01; *** = *p* < 0.001

We next investigated the effect of α-syn (auto)antibodies on the secretion of cytokines and chemokines, another important property of reactive astrocytes [35]. Interestingly, treatment with α-syn (auto)antibodies caused a significant upregulation of one particular chemokine, RANTES, in both neuron-astrocyte co-cultures (where human α-syn is expressed specifically in neurons, but secreted α-syn may be taken up by astrocytes) and astrocytes-enriched cultures (where human α-syn is expressed in astrocytes) (Fig. 5A, B, Suppl. Figure S10A, B). No RANTES secretion was detectable in co-cultures with neurons expressing only NmC, or in astrocytes expressing only eGFP after treatment with serum containing α-syn AAbs. We further investigated whether α-syn (auto)antibodies-mediated increase in RANTES is toxic or protective to neurons. To this end, in neuron-astrocyte co-cultures, we either depleted freely available RANTES by adding anti-RANTES antibodies or blocked RANTES uptake by inhibiting its receptors CCR1, 3, and 5. Interestingly, the application of serum containing α-syn AAb together with anti-RANTES antibody or after blockade of all RANTES receptors further decreased the number of NmC positive cells, indicating a rather protective role of RANTES (Fig. 5C, D). However, recombinant RANTES did not rescue the neuronal cell death caused by serum containing α-syn AAb (Suppl. Figure S11). This might have been due to insufficient biological activity of the recombinant RANTES, as the protein requires appropriate oxidation of cysteines to achieve the correct pattern of conserved disulphide bonds [36].

### α-synuclein autoantibodies cause neuronal cell death by NMDA receptor-mediated calcium influx

**Fig. 6:**
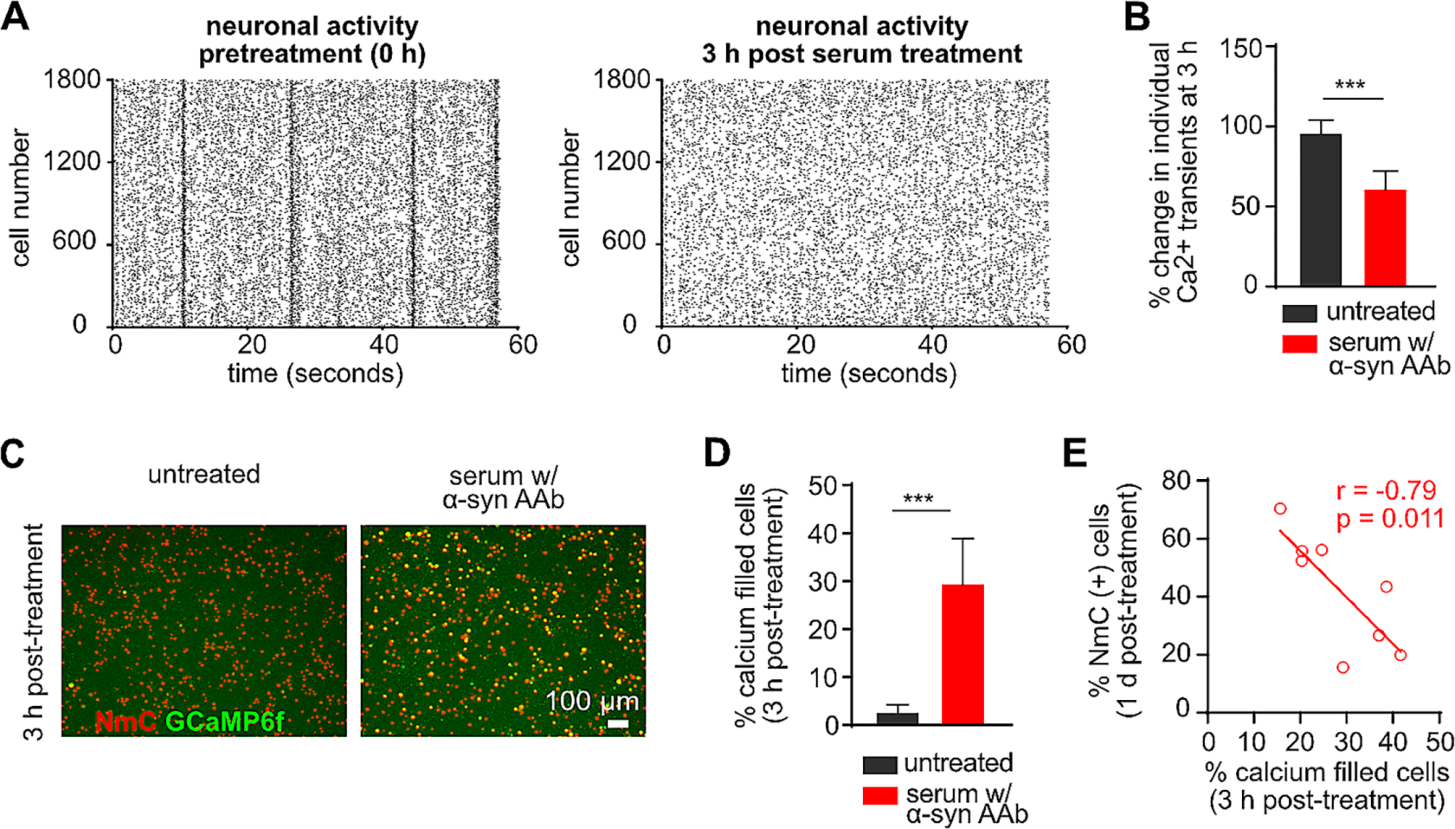
Abrogated network activity and elevated calcium influx precede neurotoxicity caused by serum containing α-syn AAb. **(A)** Scatter plot of electrical activity of individual neurons (in neuron-astrocyte co-culture at div 14), showing loss of synchronized activity after 3 h of serum treatment. Each dot represents a calcium transient as a surrogate for action potentials, while vertical lines represent non-stimulated, coordinated network activity, as recorded by the GCaMP6f sensor. **(B)** Quantification showing a significant decrease in non-synchronous transients at 3 h after serum treatment as compared to the untreated condition. **(C)** Representative live cell images of neuron-specific NmC and GCaMP6f expression in α-syn + NmC overexpressing cells, in untreated control and after 3 h of treatment with serum containing α-syn AAbs. **(D)** Quantification of percent calcium-filled cells (relative to NmC-labelled neurons) at 3 h post-serum treatment as compared to the untreated condition. **(E)** Scatter plot with correlation coefficient (r) and associated significance (p) showing a significant negative dependence between percent calcium-filled cells after 3 h and percent NmC positive cells after 1 d of treatment with serum containing α-syn AAbs. *N* = 4–5 biological and 8–9 technical replicates. Statistics by unpaired Student’s T-test; statistical power (1-ß error probability) > 0.9 for all conditions. *** = *p* < 0.001

We next explored the underlying mechanism of the neurotoxic effect of α-syn (auto)antibodies on primary neurons. To this end, we investigated neuronal survival and neuronal activity at an early time-point of 3 h after treatment with serum containing α-syn autoantibodies. We did not observe any neurodegeneration at this time point (Suppl. Figure S12A). However, a complete loss of non-stimulated, synchronized network activity (Fig. 6A, B, Suppl. Figure S12B) and a 40% decrease in single spiking events (Fig. 6A, B) was detected via calcium imaging at 3 h post serum treatment. Along with the sudden loss of neuronal activity, serum containing α-syn AAbs caused an increase in intracellular calcium (Fig. 6C). ∼30% of NmC positive neurons were found to have robustly elevated intracellular calcium levels as displayed by an irreversible GCaMP6f signal, within 3 h of serum treatment as compared to the untreated cells (Fig. 6D). Furthermore, the percentage of calcium-filled cells at 3 h showed a significant, negative correlation to the percentage of surviving neurons after 1 d of treatment with serum that contains α-syn AAb (Fig. 6E). These data suggest that serum that contains α-syn AAb caused a sudden rise in intracellular calcium thus leading to compromised neuronal survival.

**Fig. 7:**
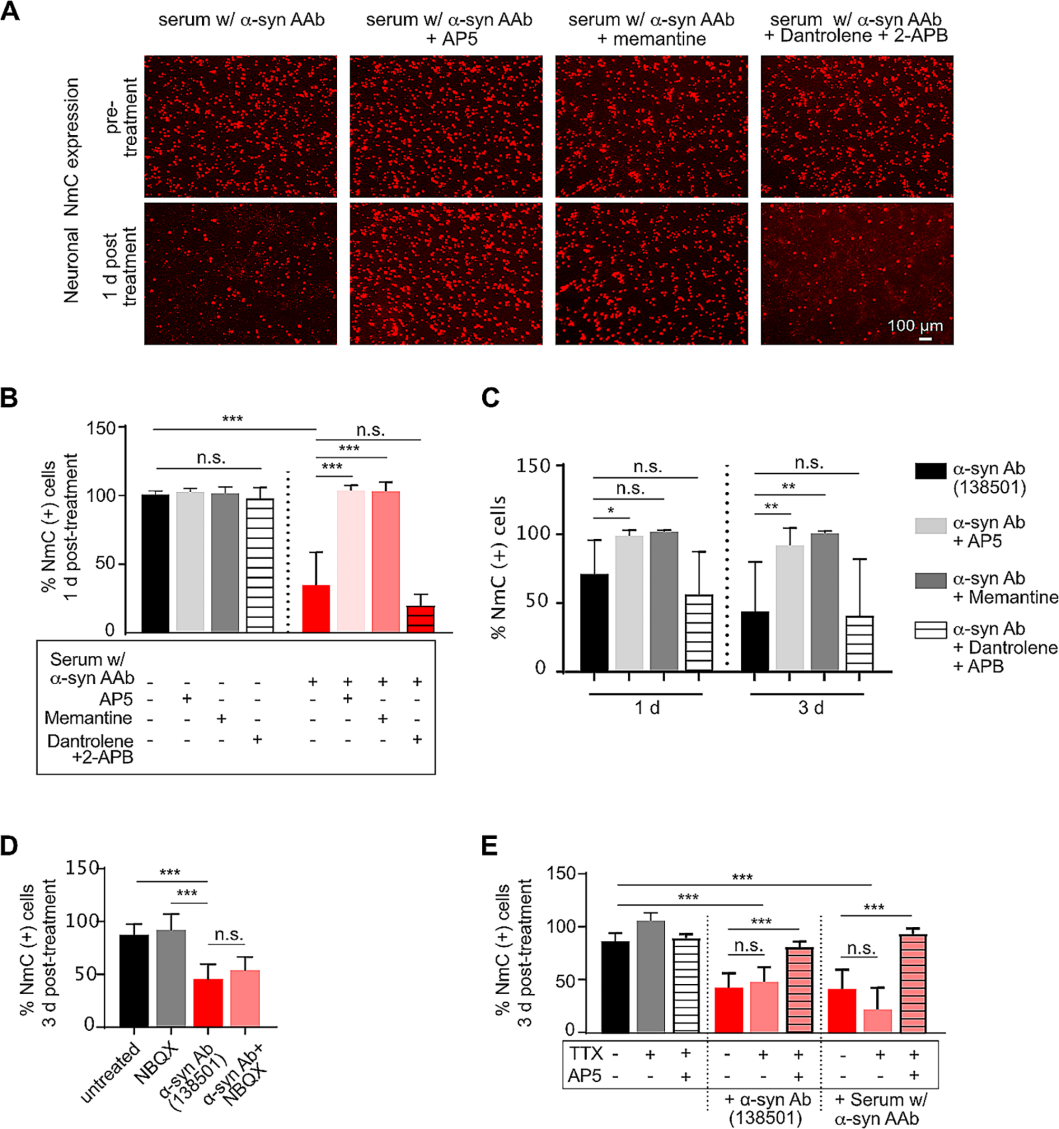
Blockade of NMDA receptors rescues neurotoxicity caused by α-synuclein (auto)antibodies. **(A)** Representative images of neuronal NmC expression in α-syn + NmC overexpressing cultures before (pre-treatment, upper panel) and after 1 d (lower panel) of treatment with serum containing α-syn AAbs in the presence or absence of NMDA-R blockers, AP5 and memantine, or with antagonists of intracellular calcium transporters RyR (dantrolene) and IP3-R (2-APB). **(B)** Quantification of percent NmC-positive cells (relative to untreated cells) in α-syn + NmC overexpressing neurons at 1 d or 3 d post-treatment with serum containing α-syn AAb, in presence of blockers of calcium channels. **(C)** Quantification of percent NmC-positive cells /relative to untreated cells) in α-syn + NmC overexpressing neurons at 1 d or 3 d post-treatment with anti-α-syn Ab (138501), in presence of blockers of calcium channels. **(D, E)** Blocking other glutamate receptors like AMPA receptor with NBQX **(D)** or a complete blockade of neuronal activity using tetrodotoxin (TTX) **(E)** does not rescue α-syn Ab-mediated neurodegeneration. *N* = 3–5 biological and 6–10 technical replicates. Statistics by one-way ANOVA with Tukey’s **(B)** or Dunnett’s **(C - E)** multiple comparison test; statistical power (1-ß error probability) > 0.9 for all conditions. * = *p* < 0.05; ** = *p* < 0.01; *** = *p* < 0.001

We next investigated the upstream mechanism responsible for increased intracellular calcium levels. Physiologically, calcium concentration is highest in extracellular space (1–2 mM). Within the cell, the endoplasmic reticulum (ER) serves as the major calcium reservoir with a concentration ranging from 100 to 800 µM as compared to 0.1 µM in the cytosol [37]. The increased cytosolic calcium seen 3 h post serum treatment can therefore be mediated either by an influx from extracellular space or by efflux from intracellular reservoirs. Therefore, we next blocked calcium release from ER to the cytoplasm by using Dantrolene and 2-APB, inhibitors for ryanodine receptors (RyR) [38] and IP3 receptors (IP3R) [39], respectively. In already depolarized neurons, activation of the postsynaptic N-Methyl-d-aspartate receptor (NMDAR) can largely mediate calcium influx from the extracellular space [37, 40]. We, therefore, blocked NMDAR by using the antagonists 2-amino-5-phosphopentanoic acid (AP5) [41] or memantine [42]. All inhibitors were applied 30 min before serum or α-syn antibody treatment. The inhibitors themselves did not cause any neurotoxicity at the concentrations used. No significant difference in neuronal survival was observed after treatment with α-syn (auto)antibodies in the presence or absence of RyR and IP3R blockers. In pronounced contrast, pre-treatment with NMDAR antagonists significantly rescued neuronal cell death post-serum treatment. A similar protective effect of AP5 and memantine was observed when the cells were treated with a purified, commercial α-syn antibody (Fig. 7A-C). These data point towards an NMDAR-mediated influx of calcium from the extracellular space into the cytosol upon interaction of aSyn AAbs or Abs, a process that inevitably causes excitotoxicity-like neurodegeneration.

Application of NMDAR blockers AP5 or memantine robustly silenced neuronal activity (Suppl. Figure S13B, C). Thus, it might be possible that the neuroprotective effect of these blockers would at least partially depend on diminished neuronal activity. Importantly, neuronal activity was also silenced by RyR and IP3 blockers (Dantrolene and 2APB), by AMPA receptor blocker NBQX and sodium channel blocker TTX (Suppl Figure S13 D – F), however, none of these treatments prevented α-syn AAb- or α-syn Ab-mediated neurodegeneration (Fig. 7D, E). Thus, acute neuronal activity itself seems not to be necessary for induction of neurodegeneration through α-syn AAb or α-syn Ab application. The subtle increase in extracellular glutamate that was detected after application of α-syn AAb or α-syn Ab to neuron-astrocyte co-cultures might thus represent an enhancing effect on neurotoxicity rather than an independent inducer of neurodegeneration (Suppl. Fig. S14).

## Discussion

Autoantibodies against α-syn, which are present in the blood of almost all individuals, were initially considered potential biomarkers for PD. Recent research has shown that α-syn AAb serum levels are indeed lower in patients with neurodegenerative diseases as compared to healthy controls, but these levels do not differ between various neurodegenerative disorders, and thus cannot distinguish between e.g., PD and AD [29]. If lower serum levels correlate with enhanced drainage of α-syn AAb into the brain through an impaired BBB remains unknown. Furthermore, serum titres of α-syn AAbs differ substantially between various cohorts of patients and controls examined thereupon [29]. α-syn AAbs were also considered to be potentially neuroprotective, as they are able to influence the aggregation state and seeding of α-syn [26], and were non-toxic when applied to α-syn-expressing cell lines [27]. In pronounced contrast to these earlier studies, we now demonstrate that α-syn AAbs can induce a robust neuropathological phenotype, characterized by NMDAR-mediated calcium influx, silencing of neuronal network activity, and subsequent neurodegeneration. This neurodegenerative effect of α-syn AAbs was detected both in primary rodent neurons and in iPSC-derived human neurons.

Should this scenario hold true for the brain of PD patients, were evidently plasma-borne IgGs are present in vulnerable brain nuclei such as SNpc and STN [22, 43], it appears plausible that α-syn AAbs that infiltrated the CNS could contribute to disease etiology. The blood-brain-barrier (BBB) is impaired in many neurodegenerative disorders including PD [44], and the extent of its leakiness may act as a critical denominator for the extent of α-syn AAb penetration, potentially contributing to the rather variable clinical phenotype and/or disease progression seen in PD patients [45].

The precise location where α-syn AAbs interact with α-syn is currently unknown. Within the CNS α-syn is a highly abundant protein, mostly located at pre-synaptic sites at the membrane of synaptic vesicles. However, α-syn-containing aggregates are found within the cytoplasm and neuropil, suggesting that the protein has also non-synaptic locations, at least during intracellular transport and/or degradation processes [46]. Furthermore, α-syn is released from neurons in an activity-depending process [47] (and see Ref 30, Fig. 3 for a detailed evaluation in cultured neurons as used in this study), suggesting that it reaches the extracellular space starting from synaptic sites. It is likely that during synaptic vesicle fusion α-syn bound to vesicle membranes is targetable by antibodies against α-syn. Binding of the antibody might then cause steric problems with vesicle recycling and the ability to release further neurotransmitters. This mechanism may explain the impact of α-syn AAbs on neuronal activity. Alternatively, or in addition, secreted α-syn might be bound by antibodies when being associated with NMDAR. Extracellularly applied α-syn in oligomeric state has been demonstrated to interact with the GluN2A subunit of NMDAR, thereby causing synaptic dysfunctions, i.e., deficits in long-term potentiation [48–50], and direct activation of extra-synaptic NMDAR by α-syn oligomers has also been described [51]. Thus, given that in our cell culture system α-syn is released from spontaneously active neurons in significant amounts [30], it appears plausible that this secreted protein can interact with NMDAR. Targeting this receptor-bound α-syn by an antibody may then induce a conformation change resulting in increased ion permeability of NMDAR, causing calcium influx and subsequent excitotoxicity-like cell death. If this happens at synaptic and/or extra-synaptic NMDAR, for which either neuroprotective or neurodegenerative properties are postulated [52], remains to be evaluated. It will also be important to investigate if and how these mechanisms apply in the brain, where synaptic sites are probably more shielded from the environment as compared to our two-dimensional cultures. Importantly, antibody-mediated targeting of α-syn can also induce neurotoxicity in absence of any ectopic overexpression of the human protein in rodent neurons, since an antibody directed specifically against rodent α-syn induced the same magnitude of neurotoxicity in rat neurons expressing only the endogenous rat α-syn.

Mechanistically, our data suggest that electrical activity of neurons might be important to release α-syn from synaptic sites, to serve as an interaction partner for NMDAR and thus as a mediating element between NMDAR and α-syn AAbs. However, acute electrical activity itself is not necessary to make neurons vulnerable to α-syn AABs, since neurons that were silenced by blockade of AMPA-R with NBQX or sodium channels with TTX were still susceptible to induction of neurotoxicity by α-syn AABs. This finding further strengthens the hypothesis that α-syn AABs act rather directly on NMDAR associated α-syn than on synaptic vesicle-associated α-syn.

Astrocytes are absolutely essential for proper brain function by establishing a functional BBB and by protecting and nourishing neurons [32]. Here, native astrocytes, either in neuron-astrocyte co-culture, or in astrocyte-enriched cultures (> 99% astrocytes), did not react to α-syn AAbs or purified, commercial α-syn antibodies. However, in the presence of α-syn, either as secreted protein in neuron-astrocyte co-culture or if expressed within astrocytes, astrocytes reacted by specifically secreting the chemokine RANTES, while other cyto- and chemokines (IFNγ, TNFα, CCC2, CXCL1, IL12) were not elevated in cell culture supernatants. Previous studies have shown a similar elevation of RANTES in the serum and mid-brain of PD patients and MPTP-treated mice [53, 54]. It remains enigmatic for the time being where and how α-syn AAbs might interact with α-syn in astrocytes and how this interaction would initiate RANTES secretion. Given that capturing of RANTES or blocking its receptors moderately enhanced the neurotoxicity of α-syn AAbs, it appears that the astrocytic reaction is rather a neuroprotective attempt. However, if and how α-syn AAbs could induce a complex interplay of neurotoxic mechanisms through NMDAR activation on neurons and neuroprotective signaling of astrocytes in human brain remains to be determined. None the less, our data suggest that the individual extent of α-syn secretion, BBB leakiness and α-syn AAbs plasma levels may significantly influence the neurodegenerative events in patients affected by synucleinopathies, adding evidence that these parameters might be important co-factors for the wide spectrum of disease manifestations seen in clinical practice.

Our work is also of importance for another field, that has gained considerable interest recently, i.e., immunotherapies targeting α-syn aggregation and propagation [13]. Our data strongly suggest that antibodies directed against α-syn may cause remarkable side-effects if they reach the CNS via a leaky BBB, a view supported by recent work showing that mice actively immunized against a C-terminal peptide of α-syn demonstrated neurodegeneration at least under conditions of α-syn overexpression in the target tissue [55].

## Conclusion

This study demonstrates that α-syn AAbs, which are present in plasma of almost all individuals, can induce neurodegeneration by aberrant NMDAR activation. This finding adds a naturally occurring entity, plasma-borne anti-α-syn IgGs, to the list of culprits potentially involved in etiology and/or progression of Parkinson´s disease and other synucleinopathies. The quantitative contribution of α-syn AAbs to pathology in synucleinopathies would probably depend on the extent of BBB impairment (and thus cerebro-vascular pathology) of the individual patient, thereby offering an explanation for the heterogeneity of clinical phenotypes seen in these diseases.

## Materials and methods

### Serum samples

As described previously [29], serum samples from idiopathic PD patients and age- and gender-matched healthy subjects were obtained from the Department of Neurology, University Medical Center, Göttingen. All patients underwent detailed neurological and neuropsychological examinations by experienced movement-disorder specialists. PD patients were diagnosed according to the criteria of the International Parkinson and Movement Disorder Society [56]. All participants provided informed consent and the study protocols (Nr. 13/11/12 and 4/5/21) were pre-reviewed by the Institutional ethics committee in concordance with the Declaration of Helsinki.

### Enzyme linked immuno-sorbant assay (ELISA)

The concentration of α-synuclein (α-syn) autoantibodies (AAb) in the serum was measured using ELISA as described previously [29]. Briefly, high-binding 96-well plates coated with 20 ng/ml of α-syn were blocked with 5% bovine serum albumin (BSA) for 1 h at room temperature (RT). Serial dilutions of the pan-synuclein antibody (ab6176) were used as a standard. Standards and 1:500 diluted samples were incubated for 2 h at RT, followed by incubation with HRP-conjugated antibody and developing with TMB. The reaction was stopped with 1 M sulfuric acid and read at 450 nm. The detection range of our assay was 7-500 ng/ml with 4.5% and 12% intra- and inter-assay coefficients of variation respectively. Uncoated wells were used as negative controls.

### Depletion of α-synuclein autoantibodies

Recombinant α-synuclein (α-syn) protein with 6X His-tag was synthesized as described previously [57]. Subsequently, the α-syn protein was bound to nickel-charged nitrilotriacetic acid (Ni-NTA) magnetic beads (catalog no. 88832) in a buffer containing 10 mM Imidazole, 50 mM NaH_2_PO_4_ and 300 mM NaCl, pH 8.35 for 2 h with continuous rotation. Beads were then washed two times to remove unbound α-syn protein. Subsequently, α-syn AAb containing serum was incubated with α-syn-bound magnetic beads for 4–5 days with continuous rotation. Every two days, old beads were replaced with beads freshly bound to α-syn protein to allow maximum depletion of α-syn AAbs from the serum. Lastly, a strong magnetic field was applied to separate beads from the serum. This was repeated thrice with 5–8 min of incubation each time to ensure the complete removal of beads from the serum (Fig. 2A). Serum was maintained sterile and cold (4–8 °C) at all steps. As confirmed by ELISA, this method allowed up to 80–90% depletion of α-syn AAbs from the serum.

### Adeno-associated viral (AAV) vectors

All vectors were generated using the AAV-6 serotype. This serotype can transduce both neurons and astrocytes. Strictly neuron- or astrocyte-specific transgene expression was achieved by using either the synapsin1 or the full length GFAP promoter [33, 58]. AAVs were generated in transiently transfected HEK293 cells and purified by iodixanol gradient centrifugation, and heparin affinity chromatography. Following purification, FPLC eluates were dialyzed against PBS, aliquoted, and frozen at -80^o^C. The titer of vector genomes (vg) was determined by qPCR. The number of transducing units (tu) was calculated based on the experimentally determined 1:30 (tu:vg) ratio.

### Cell culture

**Rat primary neuron-astrocyte mixed cultures** were prepared from embryonic day 18 (E18) rat brain cortices as described previously [58]. Briefly, the cortices were excised, enzymatically treated, and triturated. Cells were plated at a density of 250,000 cells per well in 24-well plates coated with 0.1 mg/ml poly-L-ornithine and 1 µg/ml laminin. Cultures were maintained in the supplemented neurobasal medium at 37 °C, 5% CO_2_, and 95% humidity. The medium was replaced once during the first week of culture while transducing with α-synuclein-expressing AAV vector. The medium was not changed further to allow the accumulation of released synuclein. Cells were transduced with AAVs expressing Bcl-XL to avoid toxicity due to overexpressed α-synuclein.

**The astrocyte-enriched cultures** were generated as described previously [59]. Briefly, astrocytes from the triturated cortices the of E18 rat brain were targeted with a biotinylated-anti-GLAST antibody and captured with an anti-biotin antibody bound to magnetic beads. The cell suspension mixed with antibodies was passed through a column attached to a strong magnetic field. This allowed the selection of antibody-bound astrocytes while other cell types passed in the flow-through. Astrocytes were collected by detaching the column from the magnetic field. Cells were maintained in a serum-free medium containing DMEM-F12, Penstrep, Glutamax, 1X B27 without retinoic acid, and 0.5X N2 supplement. Enriched astrocytes were used for a maximum of 5 passages.

**iPSC-derived glutamatergic neurons** were generated as described previously [60]. Briefly, CT-01 hiPSC line from a healthy subject was plated on matrigel coated wells and cultured for 12 days. The neural induction medium consisted of knockout DMEM medium, 15% knockout serum replacement and 2 mM L-Glutamine that was gradually changed to Neurobasal, 1% B27 and 2 mM L-Glutamine. For neural induction, dual SMAD inhibition was applied using LDN193189 (1 µM) and SB432545 (10 µM) from day 1 to 7. FGF-2 (10 ng/ml; Peprotech; 100-18B) was added to the medium from day 3 to 12 to expand the neural progenitors. At day 13, the cells were dissociated using accutase and re-plated with rat astrocytes in wells pre-coated with poly-L-ornithine and laminin. The cells were maintained in the maturation medium until analysis. The maturation medium consisted of Neurobasal, 1% B27, 2 mM L-Glutamine, 20 ng/ml BDNF, 20 ng/ml GDNF and 10 µM DAPT.

### Transductions and treatments

Rat neuron-glia co-cultures were transduced with AAV vectors expressing low levels of Bcl-XL, to prevent any neurotoxicity caused by α-synuclein expression [30, 31], and the genetically encoded calcium sensor GCaMP6f [30] to record neuronal activity, on day in vitro (DIV) 2 (3 × 10^7^tu/well). Both transgenes were expressed from the strictly neuron-specific synapsin1 promoter. On DIV4, cells were transduced with AAV vectors expressing either the fluorophore nuclear-targeted mCherry (NmC) alone or with bi-cistronic vectors expressing human α-synuclein + NmC (2 × 10^8^tu/well) (Fig. 1A). These transgenes were also expressed under the synapsin1 promoter to allow neuron-specific expression. On DIV14, cells were treated with serum containing α-synuclein autoantibodies or with commercial antibodies. For experiments with human iPSC-derived glutamatergic neurons, 1 day after plating, the cells were transduced with AAV-GCaMP6f (1 × 10^8^ tu/well). Two days later, full medium change was performed and cells were transduced with neuron-specific AAV-α-synuclein + NmC (2 × 10^8^tu/well). After 30 days of culture, human neurons were treated with serum containing α-synuclein autoantibodies or with commercial antibodies. Human neurons were cultured for 30 days before being used for experiments as they acquired a similar spontaneous activity at a later time-point as compared to the rat neurons.

For a similar treatment of astrocyte-enriched cultures, cultures were transduced with AAV vectors expressing enhanced green fluorescent protein (AAV-GFAP-eGFP, at 3 × 10^7^tu/well) either alone or in addition with human α-synuclein, under the control of glial fibrillary acidic protein (GFAP) promoter (AAV-GFAP- α-syn, 2 × 10^8^tu/well) (Supplementary Fig. 5A). Treatments with depleted serum and commercial antibodies were performed as described above. All commercial antibodies (GAPDH Ab-G8795, GFP Ab-1181446001, Synapsin1 Ab-106011, and α-syn Abs-ab138501, 32-8100 Invitrogen, and sc7011) were applied at a concentration of 1 µg/ml. The expression of NmC in neurons and eGFP in astrocytes was used to determine cell survival after treatments, in live imaging microscopy.

100 ng/ml of anti-RANTES antibody (MAB678) was applied to sequester the secreted RANTES. BIX513 hydrochloride, SB328437, and DAPTA (all at 1 µM) were applied to block RANTES receptors, CCR1, CCR3, and CCR5 respectively. Recombinant RANTES (RnD Systems) was used at a concentration of 10 µg/ml. To block NMDA receptors (NMDAR), cells were pretreated for 30 min with 100 µM AP5 or 30 µM memantine followed by serum application. 100 nM Dantrolene and 50 µM 2-APB were used to block ryanodine and IP3 receptors, thereby inhibiting the release of calcium ions from the endoplasmic reticulum to the cytoplasm. AMPA receptor (AMPA-R) and sodium channels were blocked by 30 min of pretreatment with 10 µM NBQX and 1 µM tetrodotoxin (TTX), respectively.

### Measurement of cell survival and spontaneous network activity

To investigate the number of NmC-expressing cells and spontaneous network activity, imaging was performed as described previously [30]. Briefly, cells were imaged at 37^o^C and 5% CO_2_ with a Zeiss 5x Fluor objective (0.25 aperture) using a Zeiss Observer Z1 microscope. The same field of view was used to acquire NmC images and to record changes in GCaMP6f fluorescence for 1 min. For analysis, nuclear mCherry (NmC) was used to locate the nuclei to mark the region of interest (ROI). The number and location of cells were obtained using a custom-made Image J macro to segment the nuclear mCherry images. Calcium influx events were identified by analyzing changes in the signal of the calcium sensor using the FluoroSNNAP software. A minimum threshold of 10% of NmC-expressing neurons undergoing a calcium influx was used to identify network bursts and the percentage of active cells. This final step was performed either manually using Microsoft Excel or using a python script. To analyze the percent of calcium-filled cells (i.e. cells that showed a non-reversible increase in GCaMP6f fluorescence), a time frame without spontaneous network activity was selected from GCaMP6f recording. ROIs from segmented NmC images were used to locate the cells. The mean intensity of each ROI was determined to account for calcium-filled cells.

The number of surviving astrocytes under different conditions was determined by counting GFAP-eGFP-positive cells. 5–6 images were taken per well using a Zeiss 10X Plan-Apochromat objective (0.45 aperture) using a Zeiss Observer Z1 microscope.

### Immunostaining

Cells were washed with 1X PBS and fixed with 4% PFA for 15 min. After permeabilization with 0.25% Triton X-100 for 10 min, cells were blocked with 2% BSA and 10% NGS for 1 h to reduce the non-specific binding of antibodies. The cells were incubated overnight at 4 °C with MAP2 (ab5622) antibody. After three washes with 1X PBS, cells were incubated with an anti-rabbit secondary antibody (1:500) for 1 h. Cells were imaged with a Zeiss 20X LD Plan NeoFluar objective (0.4 aperture) using a Zeiss Observer Z1 microscope.

### Immunoblot

20 µg of cell lysates were resolved on 10% SDS-Polyacrylamide gel and transferred to the nitrocellulose membrane. After blocking with 5% milk, the membranes were incubated overnight with anti-GFAP (Z0334) and anti-vinculin (V9131) antibodies. After washing, the blots were incubated with horseradish peroxidase (HRP)-coupled secondary antibodies and developed using ECL. The intensity of the bands was determined using BioRad Image Lab software. Relative GFAP expression was calculated after normalization to vinculin intensity.

To determine any potential carry-over of the α-syn protein in the depleted serum, 500 ng of recombinant α + β + γ-syn, α-syn protein before binding, flow-through after α-syn binding, α-synAAb containing serum before and after depletion were resolved on 12% SDS-Polyacrylamide gel and transferred to the PVDF membrane. Blots were fixed with 4% Paraformaldehyde (PFA) and 0.25% Glutaraldehyde, blocked with 3% BSA and probed with a pan-synuclein antibody (32-8100 Invitrogen). Species specificity of commercial, purified antibodies, MJFR (ab138501), and D37A6 (4179), used in this study was confirmed by probing them against cell lysate from rat neuron-glia co-culture, recombinant mouse α/γ-syn and recombinant human α/β/γ-syn protein. Pan synuclein antibody (ab6176) was used as control.

### Bead-based multiplex assay

A capture bead-based assay was performed to simultaneously detect multiple cytokines and chemokines, namely IFN-γ (Interferon-γ), TNF-α, CCL2, CXCL1, IL-12 (Interleukin-12), and RANTES (Regulated on Activation, Normal T-cell Expressed and Secreted). Briefly, 1d post-treatment, the cell culture medium was collected and centrifuged at 2000 rpm at 4 °C to remove debris. Samples or standards were diluted in assay buffer. The mix was incubated with capture beads conjugated to cytokine- or chemokine-specific antibody for 2 h on a shaker at RT. Beads were collected by spinning at 1000 rpm for 5 min. After washing, a biotinylated detection antibody cocktail was incubated with the beads for 1 h. This forms a capture bead-analyte-detection antibody sandwich which was then incubated with streptavidin-phycoerythrin (SA-PE) for 30 min. The resulting fluorescence intensity of SA-PE is directly proportional to the amount of bound analytes. Beads were collected by spinning at 1000 rpm for 5 min, washed, and resuspended in wash buffer. Samples were read using FACS Canto, and BD Biosciences, and analyzed with FACS Diva software. Each analyte-specific population was segregated by size and internal allophycocyanin (APC) fluorescence of the capture beads. The concentration of each analyte was determined using a standard curve generated in the same assay.

### Glutamate measurement

The amount of glutamate in the medium 1 min post treatment with α-syn (auto)antibodies was measured using glutamate determination kit (GLN1, Sigma Aldrich) following manufacturer’s instructions. The assay measures the reduction of NAD + to NADH. It is proportional to the amount of glutamate that is oxidized to α-Ketoglutarate and ammonium ions in the presence of Glutamic dehydrogenase (GLDH) enzyme. To perform the assay, 100 µl of Tris (0.1 M)-EDTA (0.002 M)-Hydrazine buffer (64%) was added to each well for standard and samples. Then 10 µl of 30 mM NAD and 1 µl of 100 mM ATP were added in every well. Next, the required amount of water and 1 mM L-Glutamate was added to the wells for a standard curve and 89 µl of sample was added to their respective wells. The background signal was immediately measured at 340 nm using Infinite M200Pro plate reader (Tecan). Without any delay, 2 µl of L-GLDH was added to each well. The plate was covered and incubated at 22 ºC for 50 min and the change in signal was measured again at 340 nm. All the samples were assayed in duplicates to account for pipetting variation. The amount of glutamate in the medium was determined using the standard curve. Medium spiked with glutamate was used as a positive control to establish the assay.

### Statistics

Statistical analysis was performed using GraphPad Prism software. Normal distribution was assessed with D’Agostino and Pearson test. Significant differences between two groups was tested using an unpaired, two-tailed, Student’s t-test. One-way ANOVA followed by Tukey’s or Dunnet’s posthoc analysis was used to compare 3 or more groups. Correlations were tested with Pearson-rank correlation. Power analysis was performed using G*Power software (version 3.1.9.4). Achieved power was determined using a two-tailed t-test for the difference between two independent means with a posthoc analysis considering sample size for each group, effect size, and α error of probability (0.05). All bar graphs represent mean ± standard deviation. A *p*-value < 0.05 was regarded as significant after a power analysis with 80% stringency.

### Electronic supplementary material

Below is the link to the electronic supplementary material.

Supplementary Material 1

Supplementary Material 2

## Data Availability

All data generated or analysed in this study are included in this published article and its supplemental information files.

## Abbreviations

2-APB: 2-aminoethoxybiphenyl borate
AAB: Autoantibody
AAV: Adeno-associated virus
Ab: Antibody
AD: Alzheimer´s disease
α-syn: Alpha-synuclein
AP5: 2-amino-5-phosphopentanoic acid
BBB: Blood-brain-barrier
Bcl-XL: B-cell lymphoma extra large
CCL2: Chemokine (C-C motif) ligand 2
CCR1, -3, -5: C-C chemokine receptor type 1, -3, -5
CNS: Central nervous system
CSF: Cerebro-spinal fluid
CXCL1: Chemokine (C-X-C motif) ligand 1
DIV: Day in vitro
eGFP: Enhanced green fluorescent protein
ER: Endoplasmic reticulum
ELISA: Enzyme-linked immunosorbent assay
GAPDH: Glycerinealdehyde-3-phosphate dehydrogenase
GFAP: Glial fibrillary acidic protein
GluN2A: Glutamate (NMDA) receptor subunit epsilon-1
IgG: Immunoglobulin
IFNγ: Interferone gamma
IL12: Interleukin 12
IP3R: Inositol-3-phosphate receptor
NBQX: 2,3-Dihydroxy-6-nitro-7-sulfamoyl-benzo[*f*]chinoxalin-2,3-dion
NGS: Normal goat serum
NMDAR: N-methyl-D-Aspartat receptor
NmC: Nuclear-targeted mCherry
NMF: Non-motor fluctuation
NMS: Non-motor symptom
PD: Parkinson´s disease
RANTES: Regulated on activation, normal T cell expressed and secreted
RyR: Ryanodine receptor
SNpc: Substantia nigra pars compacta
STN: Subthalamic nucleus
TNFα: Tumor necrosis factor alpha
TTX: Tetrodotoxin
