# Supplementary figures and images for: Human serum-derived α-synuclein auto-antibodies mediate NMDA receptor-dependent degeneration of CNS neurons
Source: J Neuroinflammation. 2024 Feb 28;21:62. doi: 10.1186/s12974-024-03050-6 (PMC10902935; doi:10.1186/s12974-024-03050-6)

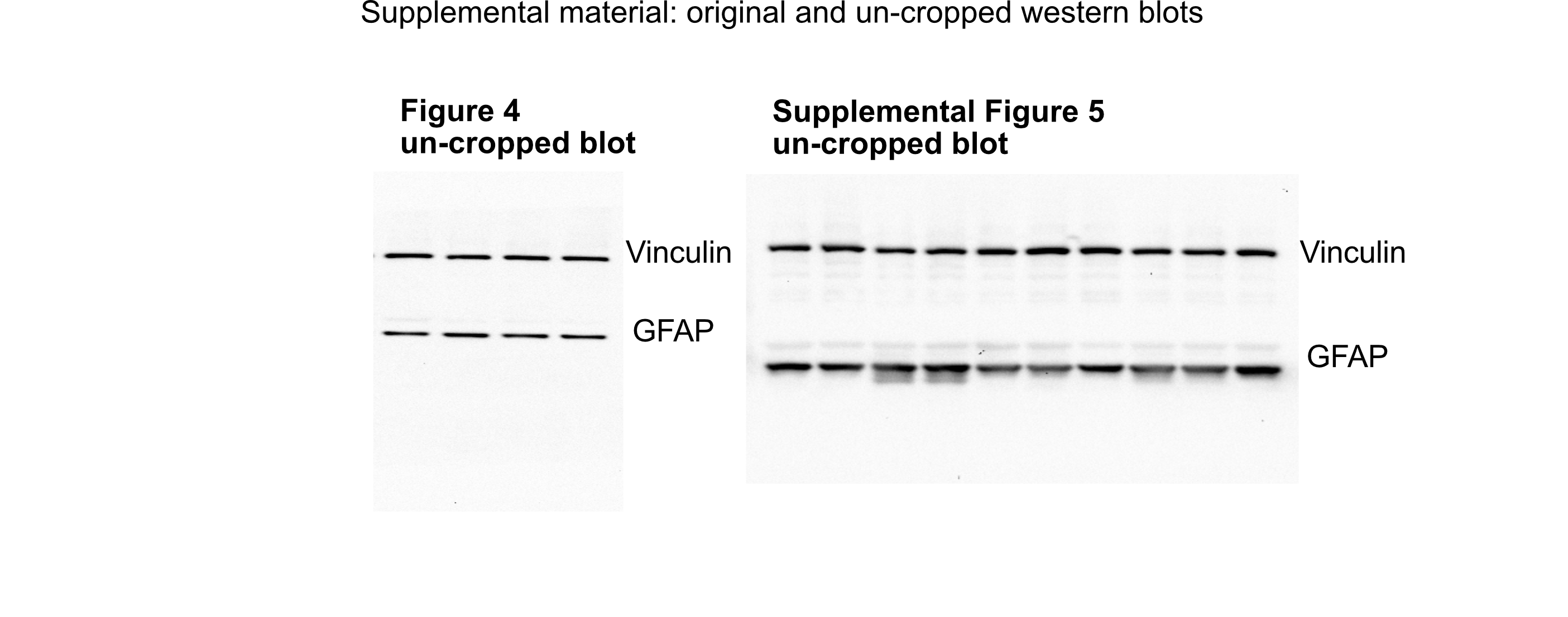

Supplement: Supplementary file 1 — Supplementary Material 1 [file 12974_2024_3050_MOESM1_ESM.png]
