# Supplementary material for: Human serum-derived α-synuclein auto-antibodies mediate NMDA receptor-dependent degeneration of CNS neurons
Source: J Neuroinflammation. 2024 Feb 28;21:62. doi: 10.1186/s12974-024-03050-6 (PMC10902935; doi:10.1186/s12974-024-03050-6)
Supplement: Supplementary file 2 — Supplementary Material 2 [file 12974_2024_3050_MOESM2_ESM.docx]

**Supplemental Figures S1 – S14 for “Human serum-derived α-synuclein auto-antibodies mediate NMDA receptor-dependent degeneration of CNS neurons” by Pretty Garg, Franziska Würtz, Fabian Hobbie, Klemens Buttgereit, Abhishek Aich, Kristian Leite, Peter Rehling, Sebastian Kügler and Mathias Bähr**


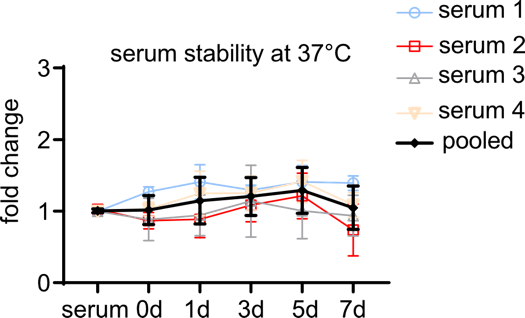


**Supplemental Figure S1. α-syn-AAbs remain stable in cell culture medium at 37°C for up to 7 d.**

The stability of α-syn AAbs in the serum at 37°C was tested by performing ELISA. The serum-treated medium was collected at 0, 1, 3, 5, and 7 d. ‘serum’ on the X-axis denotes the sample diluted in the media, without any incubation at 37°C. Serum 1-4 refers to 4 different serum samples tested with 4 technical replicates each. The black line indicates pooled data from all 4 serum samples. No significant change in the levels of α-syn AAbs was detected until 7 d.


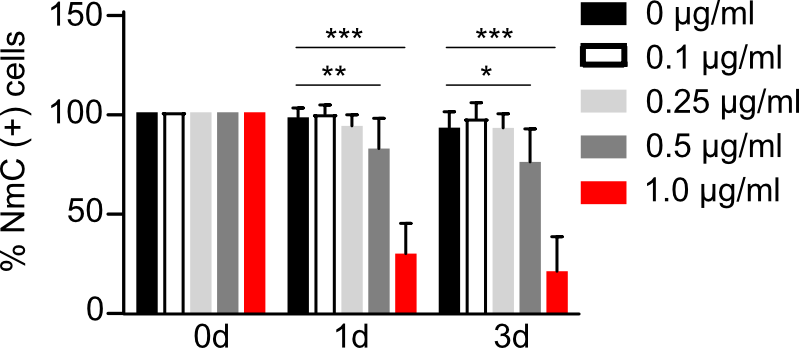


**Supplemental Figure S2.** **Dose dependency of neurodegeneration depending on concentration of α-syn AAbs.**

Percent NmC-positive cells at 0 d, 1 d and 3 d after adding serum in amounts resulting in 0, 0.1., 0.25, 0.5 and 1 µg/ml of α-syn AAbs. N = 4 biological replicates. Statistics by One-way ANOVA, followed by Dunnett’s posthoc analysis. Statistical power (1-β error probability) = 1.0 for ***, 0.72 for **, and 0.68 for *. * = p < 0.05; ** = p < 0.01; *** = p < 0.001.


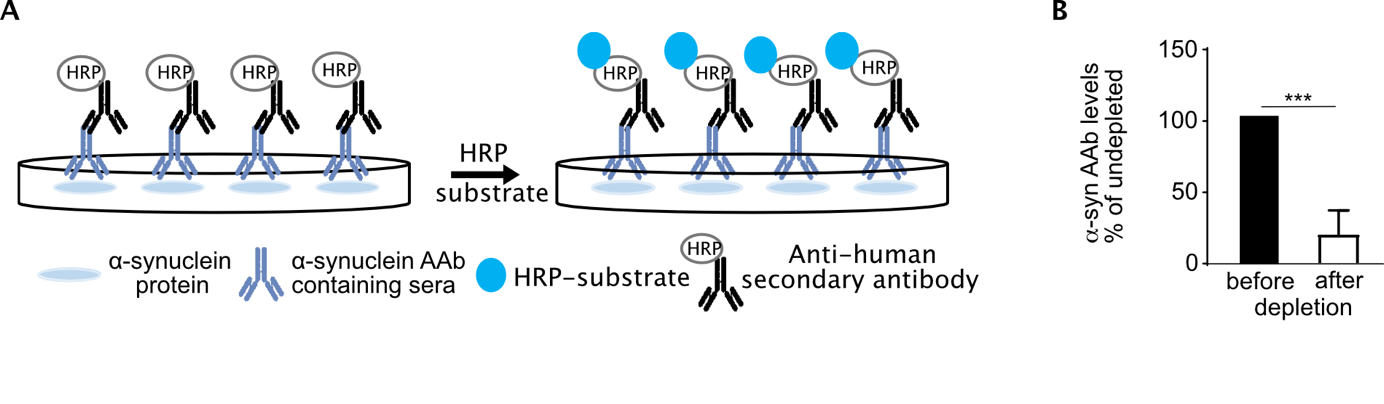


**Supplemental Figure S3. Depletion of α-syn AAbs from serum by ELISA**.

**(A)** Schematic representation of ELISA-based measurement of the levels of α-syn autoantibodies in the serum. **(B)** Levels of α-syn autoantibodies before and 6 days after depletion. N = 3 biological replicates. Statistics by Student’s t-test. Statistical power (1-β error probability) = 1.0 for ***. *** = p < 0.001.

**
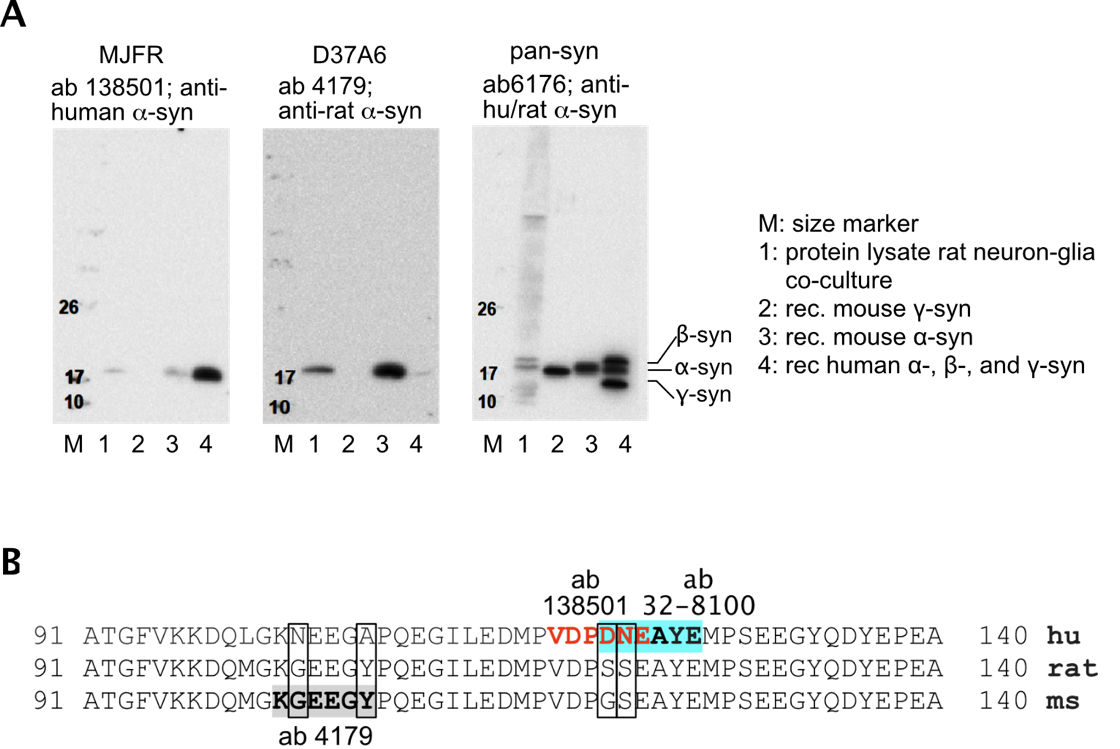
**

**Supplemental Figure S4. Specificity of purified, commercially available synuclein antibodies.**

**(A)** Western blots for different purified, commercially available anti-α-synuclein antibodies, MJFR (ab138501), and D37A6 (4179). All antibodies were probed against whole cell lysare of rat neuron-astrocyte co-culture, recombinant mouse γ- and α-synuclein, and recombinant human α+β+γ synuclein protein. Pan synuclein antibody (ab6176) with an epitope at the highly conserved N-terminal was used as a control. **(B)** The sequence of human (hu), rat, and mouse (ms) α-synuclein protein from amino acid 91-140. The known epitopes for the antibodies used are highlighted or differently colored, eg: KGEEGY for ab 4179, VDPDNE for ab 138501, and DNEAYE for ab 32-8100. Boxes denote differences in the amino acid sequences between species.

**
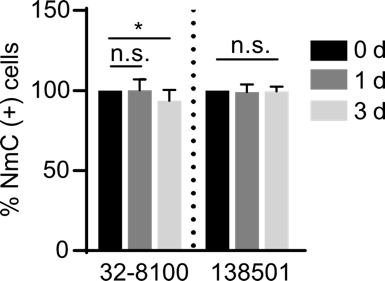
**

**Supplemental Figure S5. Purified, commercial anti-human α-synuclein antibodies cause very low or no toxicity to neurons expressing only NmC.**

Percent NmC-positive cells at 0 d, 1 d, and 3 d upon treatment with two anti-human α-syn Abs (32-8100 and 138501) at 1 µg/ml.

N = 4 biological replicates. Statistics by one-way ANOVA with Dunnett’s multiple comparisons test. Statistical power (1-ß error probability) = 0.76 for *. * = p < 0.05.


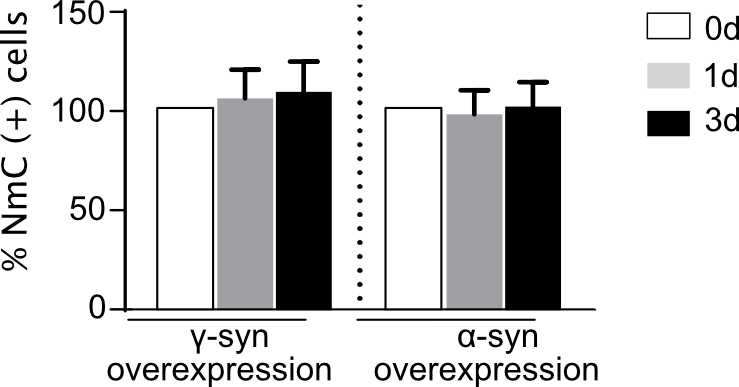


**Supplementtal Figure S6: Purified antibody against γ-syn does not cause neurodegeneration in γ-syn or α-syn overexpressing neurons at 1d or 3d of exposure.** γ-syn antibody: Ab49206. Statistics by One-way ANOVA, followed by Dunnett’s posthoc analysis.


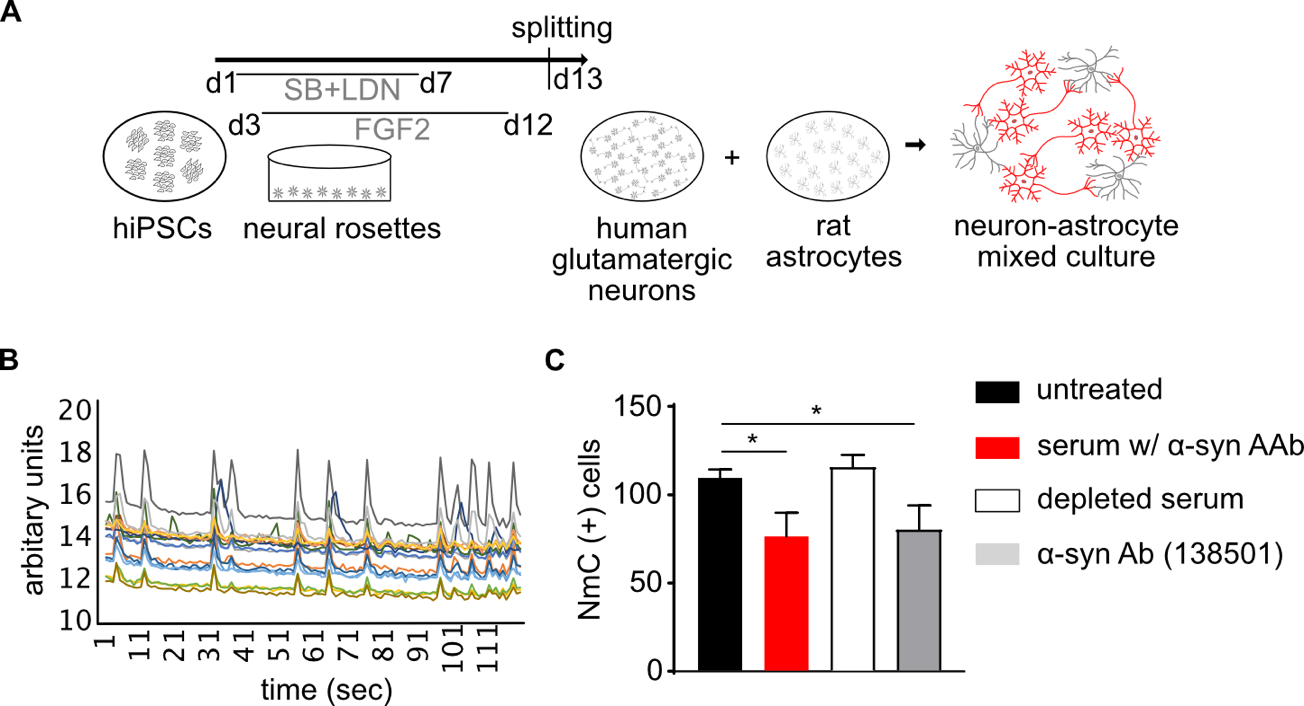


**Supplemental Figure S7:** **Effect of α-syn (auto)antibodies on human iPSC-derived glutamatergic neurons after 1d of treatment.** **(A)** Scheme of differentiation of human glutamatergic neurons from iPSCs, followed by their coculture with rat astrocytes. Co-culture with astrocytes is an absolute prerequisite for generation of spontaneous neuronal activity. **(B)** Spontaneous calcium transients at d30 of coculture of human glutamatergic neurons with rat astrocytes. **(C)** NmC positive cells after one day of treatment with serum containing α-syn AAb, with serum depleted from α-syn AAb, or with purified, commercial α-syn Ab, compared to the untreated condition. N = 2 biological replicates (independent differentiations of human neurons) and 8 technical replicates. Statistics by One-way ANOVA, followed by Dunnett’s posthoc analysis. Statistical power (1-β error probability) = 0.99 between untreated and α-syn AAb containing serum and, 0.74 between untreated and commercial α-syn Ab. * = p < 0.05.


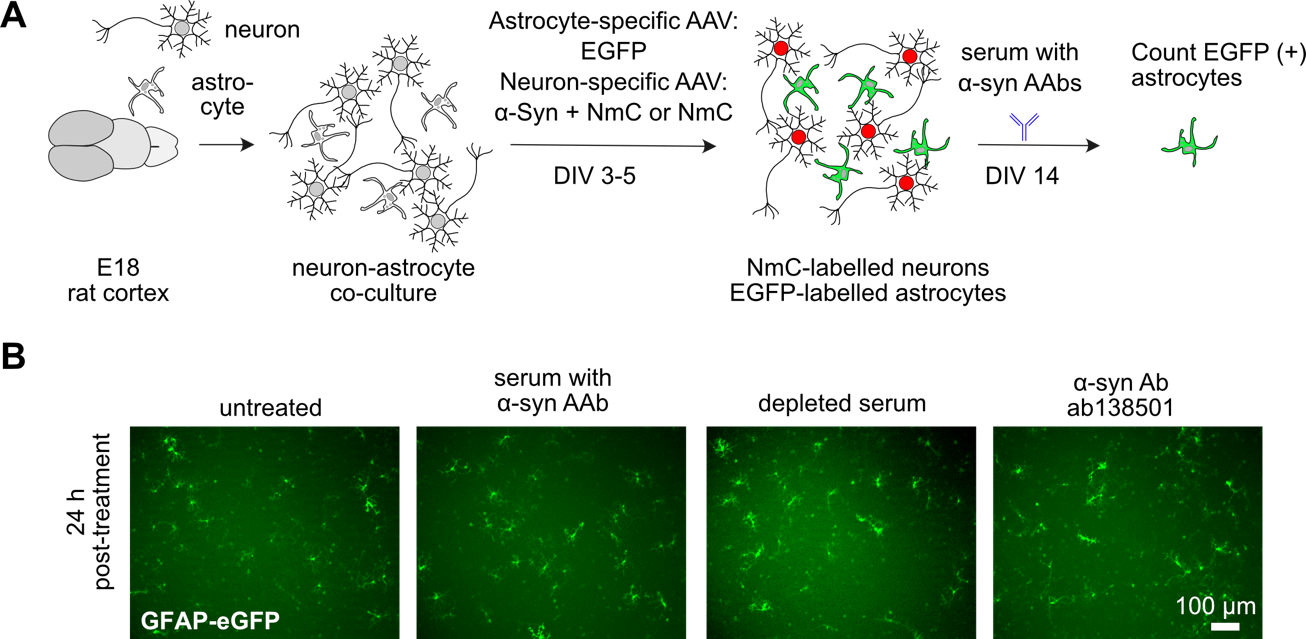


**Supplemental Figure S8. No effect of α-syn autoantibodies on astrocyte survival in neuron-glia co-culture.**

**(A)** Experimental outline showing dissociation of E18 rat cortex to generate neuron-astrocyte co-culture transduced with viral vectors encoding GFAP-driven eGFP to label astrocytes (3×10^7^tu/well). Additionally, the cells were transduced with neuron-specific AAV expressing Bcl-XL, αsyn+NmC or NmC alone. At DIV 14, cells were either left untreated or treated with serum containing α-syn AAb, serum depleted from α-syn AAb, or purified commercial α-syn Ab (ab138501). **(B)** Representative images of GFAP-driven eGFP expression in untreated controls and after 1 d of treatments.

**
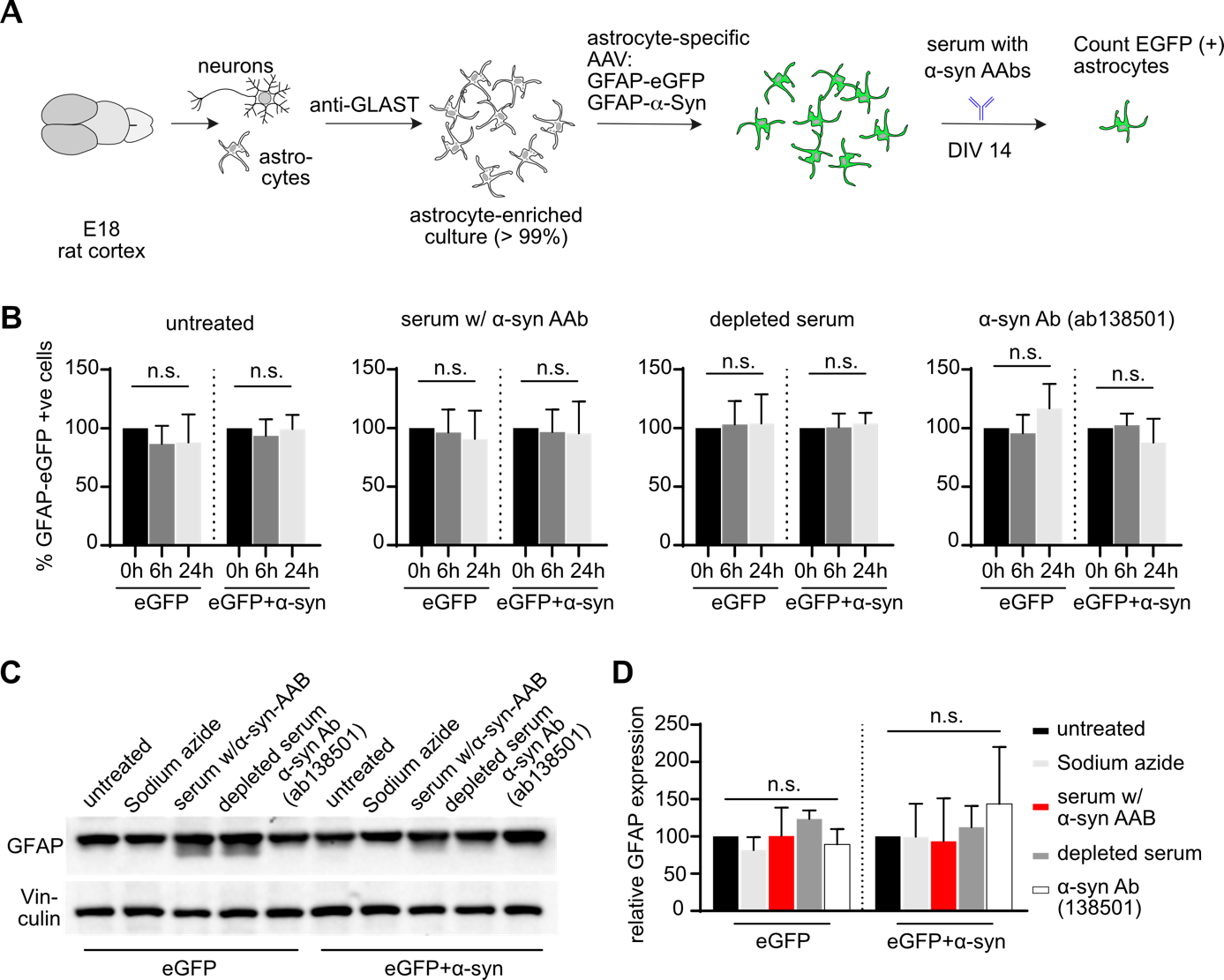
**

**Supplemental Figure S9. α-synuclein (auto)antibodies do not affect astrocyte survival in rat primary astrocyte-enriched cultures.**

**(A)** Experimental outline showing the dissociation of E18 rat cortex, followed by selection with an anti-GLAST antibody to obtain astrocyte-enriched cultures (> 99% astrocytes). Purified cells were transduced with astrocyte-specific AAV expressing eGFP (3×10^7^tu/well) alone or together with α-syn. Cells were then treated with sodium azide (0.0005%) as vehicle control or with serum containing α-syn AAb or with serum depleted from α-syn AAb, or with purified, commercial α-syn Ab (ab138501). **(B)** Percent eGFP positive cells in eGFP or α-syn+eGFP transduced cultures before treatment (0 h) and after 6 and 24 h of treatment. **(C)** Western blot for astrocyte marker (GFAP) and internal control (Vinculin) with lysates prepared from eGFP and α-syn+eGFP transduced cultures after 24 h of treatment. **(D)** Quantification of western blots as relative GFAP expression after normalization to Vinculin signal.

N = 5-7 biological replicates. Statistics by one-way ANOVA with Dunnett’s multiple comparison test


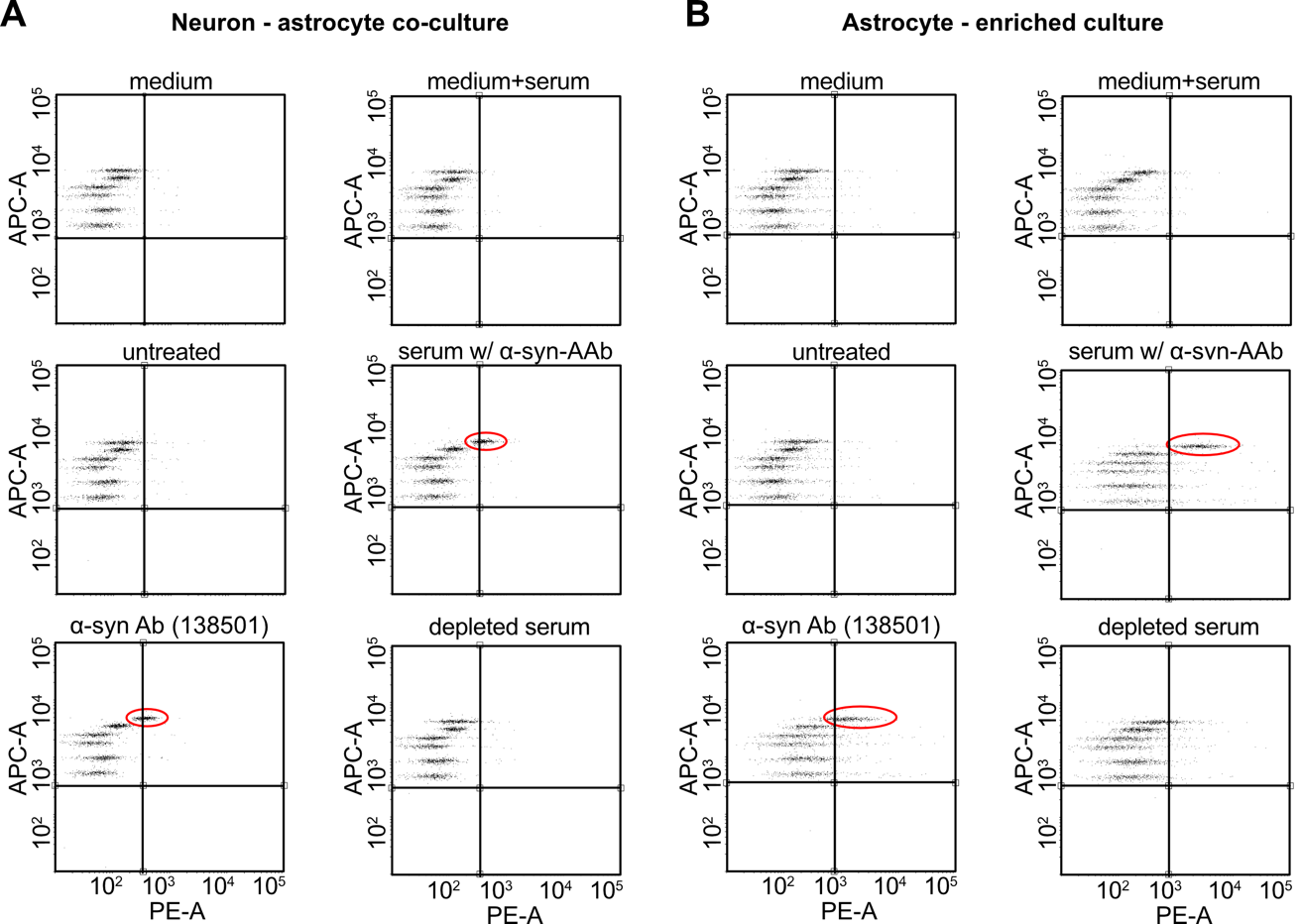


**Supplemental Figure S10. Representative graphs showing elevated RANTES upon exposure to α-synuclein (auto)antibodies.**

A shift in PE-A fluorescence showing an increase in RANTES levels (marked in red) in the presence of serum containing α-syn AAb, or commercial α-syn Ab (138501), in cell culture supernatant of neuron-astrocyte co-culture **(A)** and astrocyte-enriched culture **(B).** RANTES levels in untreated condition or upon treatment with serum depleted from α-syn AAb remained unaltered. Different clusters in APC-A fluorescence depict other tested cytokines (IFN-γ, TNF-α, CCL2, CXCL1, and IL-12) that were unchanged upon any treatment.


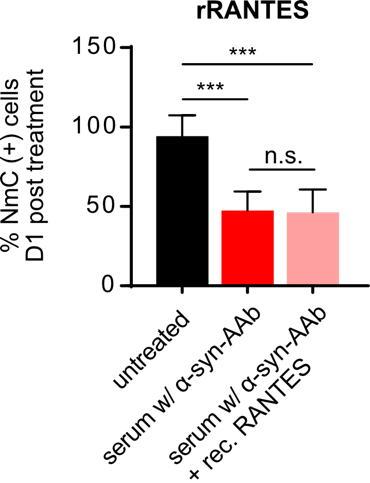


**Supplemental Figure S11. Co-treatment with recombinant RANTES does not rescue neurotoxicity caused by serum containing α-syn AAb.**

Percent NmC positive cells after 1 d of treatment with serum containing α-syn AAb, in the presence or absence of recombinant RANTES (rRANTES).

N = 4 biological replicates. Statistics by one-way ANOVA with Dunnet’s multiple comparison test; statistical power (1-ß error probability) > 0.9 for all conditions. *** = p < 0.001

**
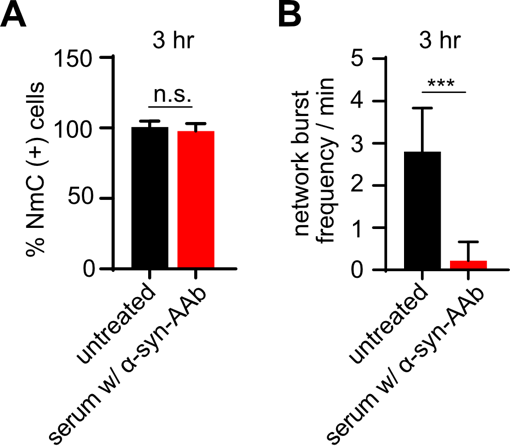
**

**Supplemental Figure S12. Abrogation of neuronal network activity within 3 hrs of exposure to serum that contains α-syn AAb.**

**(A)** Quantification of percentage NmC positive cells and **(B)** non-stimulated coordinated network bursts per minute after 3 hr of treatment with serum containing α-syn AAb.

N = 4 biological replicates. Statistics by Student’s T-test; statistical power (1-ß error probability) > 0.9. *** = p < 0.001

**
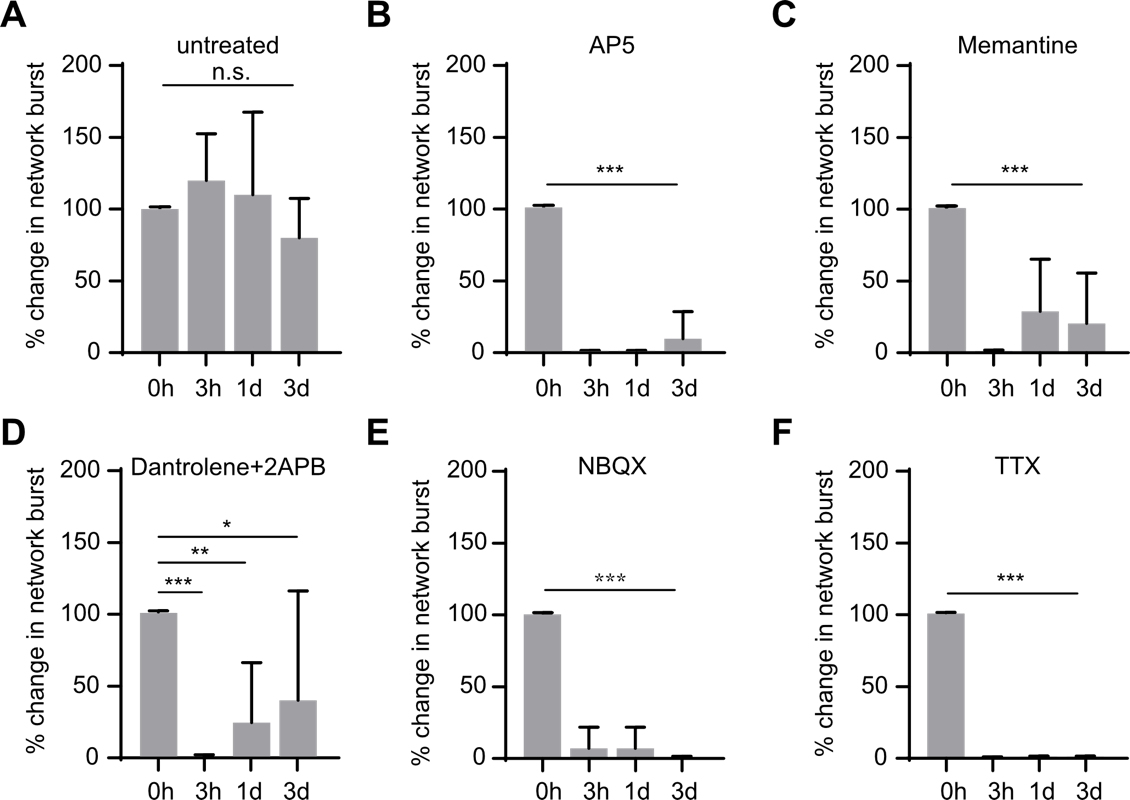
**

**Supplemental Figure S13. Abrogation of neuronal network activity by channel blockers.**

Quantification of percentage change in non-stimulated, coordinated neuronal network burst frequency in: **(A)** untreated controls, **(B)** upon blocking NMDA receptor with AP5, **(C)** upon blocking NMDA receptor with Memantine, **(D)** upon blocking endoplasmic reticulum calcium channels ryanodine and IP3 with Dantrolene and 2-APB, **(E)** upon blockade of AMPA receptors with NBQX, and **(F)** upon blockade of sodium channels with tetrodotoxin (TTX), for 0h, 3h, 1d and 3d.

N = 4 - 5 biological replicates for (A – E) and 3 biological replicates for (F). Statistics by one-way ANOVA with Dunnett’s multiple comparison test; statistical power (1-ß error probability) > 0.9 for all conditions, except * in (D), which is 0.5. * = p < 0.05; ** = p < 0.01; *** = p < 0.001.


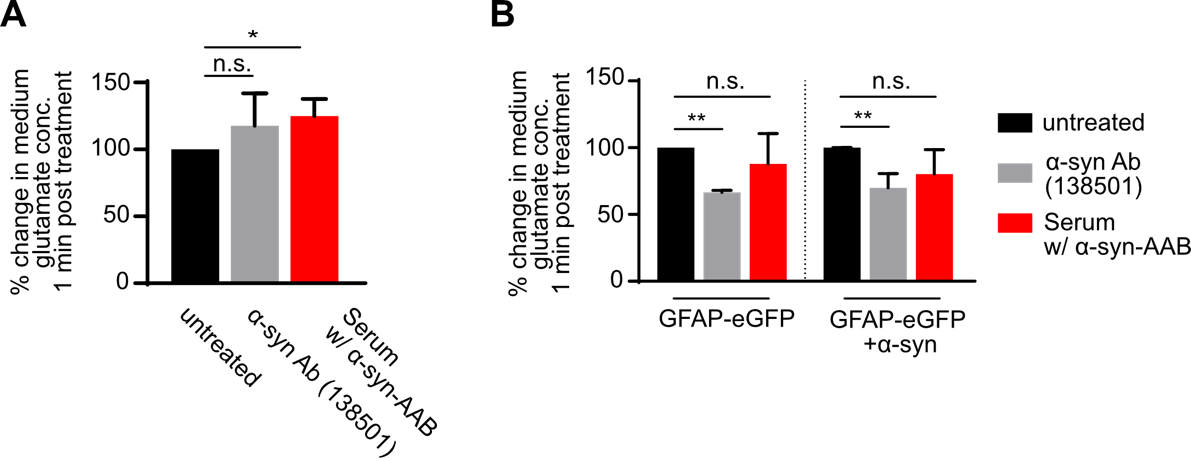


**Supplemental Figure S14. Impact of exposure to α-syn (auto)antibodies on medium glutamate concentration.**

**A)** Percent change in the medium glutamate concentration of neuron-astrocyte co-culture after 1 minute of treatment with α-syn (auto)antibodies. Cells expressed NmC in neurons. **(B)** Percent change in the medium glutamate concentration of astrocyte-enriched culture after 1 minute of treatment with α-syn (auto)antibodies. The astrocytes expressed either eGFP or eGFP+α-syn. N = 5 biological replicates for (A) and 3 biological replicates for (B). Statistics by one-way ANOVA with Dunnett’s multiple comparisons test; statistical power (1-ß error probability) > 0.9 for all conditions. * = p < 0.05; ** = p < 0.01.
